# Influence of angiotensin converting enzyme inhibitors/angiotensin receptor blockers on the risk of all‐cause mortality and other clinical outcomes in patients with confirmed COVID‐19: A systemic review and meta‐analysis

**DOI:** 10.1111/jch.14329

**Published:** 2021-07-28

**Authors:** Na Jia, Guifang Zhang, Xuelin Sun, Yan Wang, Sai Zhao, Wenjie Chi, Sitong Dong, Jun Xia, Ping Zeng, Deping Liu

**Affiliations:** ^1^ Department of Cardiology Beijing Hospital National Center of Gerontology Institute of Geriatric Medicine Chinese Academy of Medical Sciences Beijing China; ^2^ Department of Epidemiology The Key Laboratory of Geriatrics Beijing Institute of Geriatrics Beijing Hospital National Center of Gerontology Institute of Geriatric Medicine Chinese Academy of Medical Sciences Beijing China; ^3^ Department of Pharmacology Beijing Hospital National Center of Gerontology Institute of Geriatric Medicine Chinese Academy of Medical Sciences Beijing China; ^4^ Department of Respiratory and Critical Care Medicine Beijing Hospital National Center of Gerontology Institute of Geriatric Medicine Chinese Academy of Medical Sciences Beijing China; ^5^ Systematic Review Solutions Ltd Shanghai China; ^6^ Nottingham China Health Institute The University of Nottingham Ningbo China

**Keywords:** angiotensin converting enzyme inhibitor, angiotensin receptor blocker, COVID‐19, mortality

## Abstract

Since the COVID‐19 pandemic, physicians concerned about the potential adverse effects of angiotensin converting enzyme inhibitors (ACEIs)/angiotensin receptor blockers (ARBs). To explore the relationship between ACEIs/ARBs and the risk of mortality and other clinical outcomes in COVID‐19 patients, the authors conducted a systemic review and meta‐analysis. An electronic search was performed from inception to November 12, 2020 in PubMed, Medline, EMBASE, ClinicalTrials, TRIP, the Cochrane Library, CNKI, Wanfang, and CBM database. Risk of bias was assessed using the Risk Of Bias In Non‐randomized Studies of Interventions tool. The primary outcome was in‐hospital all‐cause mortality. Secondary outcomes included all‐cause mortality measured at 30‐day or longer term, mechanical ventilation, length of hospital stay, readmission, and cardiac adverse events. A total of 28 studies with 73 465 patients was included. Twenty‐two studies with 19 871 patients reported the incidence of all‐cause mortality. Results showed no association between using ACEIs/ARBs and risk of mortality crude odds ratio （OR） of 1.02, 95% CI 0.71–1.46, *p *= .90, *I* ^2 ^= 88%, adjusted OR in 6260 patients of 0.96, 95% CI 0.77–1.18, *p* = .68, *I* ^2 ^= 0%. While six studies with 10 030 patients reported a lower risk of mortality in ACEIs/ARBs group hazard ratio (HR) of 0.53, 95% CI 0.34–0.84, *p *= .007, *I* ^2 ^= 68%. Similar association (for HR) was found in hypertension subgroup. There was no significant association for the secondary outcomes. Based on the available data, we concluded that ACEIs/ARBs is not associated with the risk of in‐hospital all‐cause mortality in COVID‐19 patients, but may be associated with a decreased risk of 30‐day all‐cause mortality. Patients with hypertension may benefit from using ACEIs/ARBs.

## 1 INTRODUCTION

Since the end of 2019, COVID‐19 pneumonia erupted in many countries and became a pandemic, posing a great threat to human health and survival. SARS‐Cov‐2 can bind angiotensin converting enzyme 2 (ACE2) and allows the virus to invade cells, which may lead to activate immune response and lung injury.^1^, ^2^ ACEIs or ARBs can potentially increase ACE2 level, concern has been raised about the safety of these medications in patients with COVID‐19. Although many scientific institutions have recommended that ACEIs/ARBs therapy should not be discontinued in COVID‐19 patients,^3^, ^4^ controversial evidence has increasingly emerged regarding the effects of these medications on the prognosis of patients with COVID‐19.

In March 2020, a small sample study in China began to explore the impact of ACEIs/ARBs use on outcomes in patients with COVID‐19, with an increasing number of studies published subsequently. Most of these retrospective studies reported neutral results. Several cohort studies reported a significantly lower rate of short‐term death in ACEIs/ARBs group. To date, more than 10 published meta‐analyses have evaluated the effect of ACEIs/ARBs on mortality in patients with COVID‐19. The odds ratio (OR) showed the risk of mortality was similar for ACEIs/ARBs users and non‐users. However, these reports all included unpublished studies and most of them enrolled suspected cases. Unpublished studies did not undergo peer‐review process and may increase the risk of bias. A few of them distinguish the adjusted OR and hazard ratio (HR). Half of them only evaluated all‐cause mortality without other clinical outcomes. Therefore, we conducted a retrospective systematic review of studies evaluating patients with confirmed COVID‐19 treated with ACEIs/ARBs to further explore whether these medications influence mortality or other clinical outcomes.

## 2 METHODS

### 2.1 Literature search

This systematic review was carried out in accordance with the meta‐analysis of observational studies in epidemiology and preferred reporting items for systematic reviews and meta‐analyses statements. An electronic search was performed from PubMed, Medline, EMBASE, ClinicalTrials, TRIP, the Cochrane Library, Chinses National Knowledge Infrastructure (CNKI), Wanfang database and Chinese BioMedical (CBM) database. Drafts of protocol was developed and reviewed. The protocol was registered on PROSPERO on August 4, 2020 (CRD42020202402). Trials were identified through a comprehensive systematic search from inception to June 21, 2020 in PubMed, Medline, EMBASE, ClinicalTrials, TRIP, the Cochrane Library, CNKI, Wanfang and CBM database using the strategies presented in Appendix 1. No language restrictions were applied.

### 2.2 Selection of studies for inclusion and review

We included studies of patients with COVID‐19 confirmed by polymerase chain reaction (PCR) or genetic tests; ACEIs /ARBs use data were provided. The interventional group was ACEIs or ARBs user. The comparator was no use of ACEIs or ARBs, irrespective of combined use with other medications.

Three reviewers (NJ, XS, and YW) screened the search titles and abstracts. All potentially relevant citations were requested and inspected in detail using the full text version. Articles without full texts, duplicated publications, reports lacking data, and articles not reporting any outcomes of interest were excluded from the analysis. Disagreements were resolved by discussion with assistance from a forth reviewer (PZ).

### 2.3 Data definition and outcome assessment

Our primary outcome was in‐hospital all‐cause mortality. Secondary outcomes included all‐cause mortality measured at 30‐day or longer term, usage of mechanical ventilation, length of hospital stay, readmission and cardiac adverse events. Subgroup analysis was conducted to test the difference in effect size for the following subgroups: age (≥65‐years‐old versus  < 65‐years‐old); sex (male versus female); ACEIs or ARBs alone versus in combination with other medications; ACEIs and ARBs previously used versus ACEIs and ARBs used during hospitalization versus ACEIs and ARBs used continuously.

### 2.4 Assessment of risk of bias and reporting data

Two reviewers (SZ and WC) independently assessed the risk of bias based on methods endorsed by The Cochrane Collaboration. RoB2, the revised tool was used to assess the risk of bias in randomized trials. For cohort studies and case‐control studies, we used the Risk Of Bias In Non‐randomized Studies of Interventions (ROBINS‐I) tool to assess the risk of bias. The biases assessed include the biases associated with the randomization process, deviations from intended interventions, missing outcome data, measurement of the outcome, selection of the reported result, confounding, selection of participants into the study and classification of interventions.

### 2.5 Data extraction and management

Data from each study was extracted independently by two separate reviewers (SD and WC) using a pre‐designed data extraction form. Any disagreements were resolved by discussion with assistance from a third reviewer (SZ). We extracted all relevant characteristics of all included studies including general study characteristics, participants' information, intervention, characteristics of outcome, ACEIs/ARBs‐related risk factors with reported estimates of association and corresponding crude and adjusted estimates.

### 2.6 Statistical analysis

Meta‐analysis was performed for quantitative synthesis. In cases where data was not eligible for meta‐analysis, we narratively synthesized data. We summarized all dichotomous outcome data using odds ratios (ORs) or hazard ratios (HRs) and their 95% confidence intervals (CIs). We summarized all continuous outcome data using mean differences (MDs) and their 95% CIs. Heterogeneity in the study results included clinical heterogeneity, methodological heterogeneity, and statistical heterogeneity. When we suspected heterogeneity, we highlighted and fully discussed the reasons when this was possible. We visually inspected forest plots to investigate possible statistical heterogeneity; an *I* ^2^ estimate greater than or equal to 50% accompanied by a statistically significant chi‐square statistic was interpreted as evidence of substantial levels of heterogeneity. If the number of studies more than 10, we conducted a funnel plot to test the reporting bias of one specific outcome. We synthesized data using a fixed‐effects method for all analyses. We explored the source of heterogeneity and conducted a subgroup analysis if the source of heterogeneity was identified. We synthesized data using a random‐effects model when we could not determine the source of heterogeneity. If heterogeneity was found and the source of heterogeneity was identified, a post‐hoc subgroup analysis according to the particular factor was conducted. All analyses were performed in Review Manager 5.1 (The Cochrane Collaboration, Oxford, United Kingdom).

## 3 RESULTS

### 3.1 Review process and study characteristics

**FIGURE 1:**
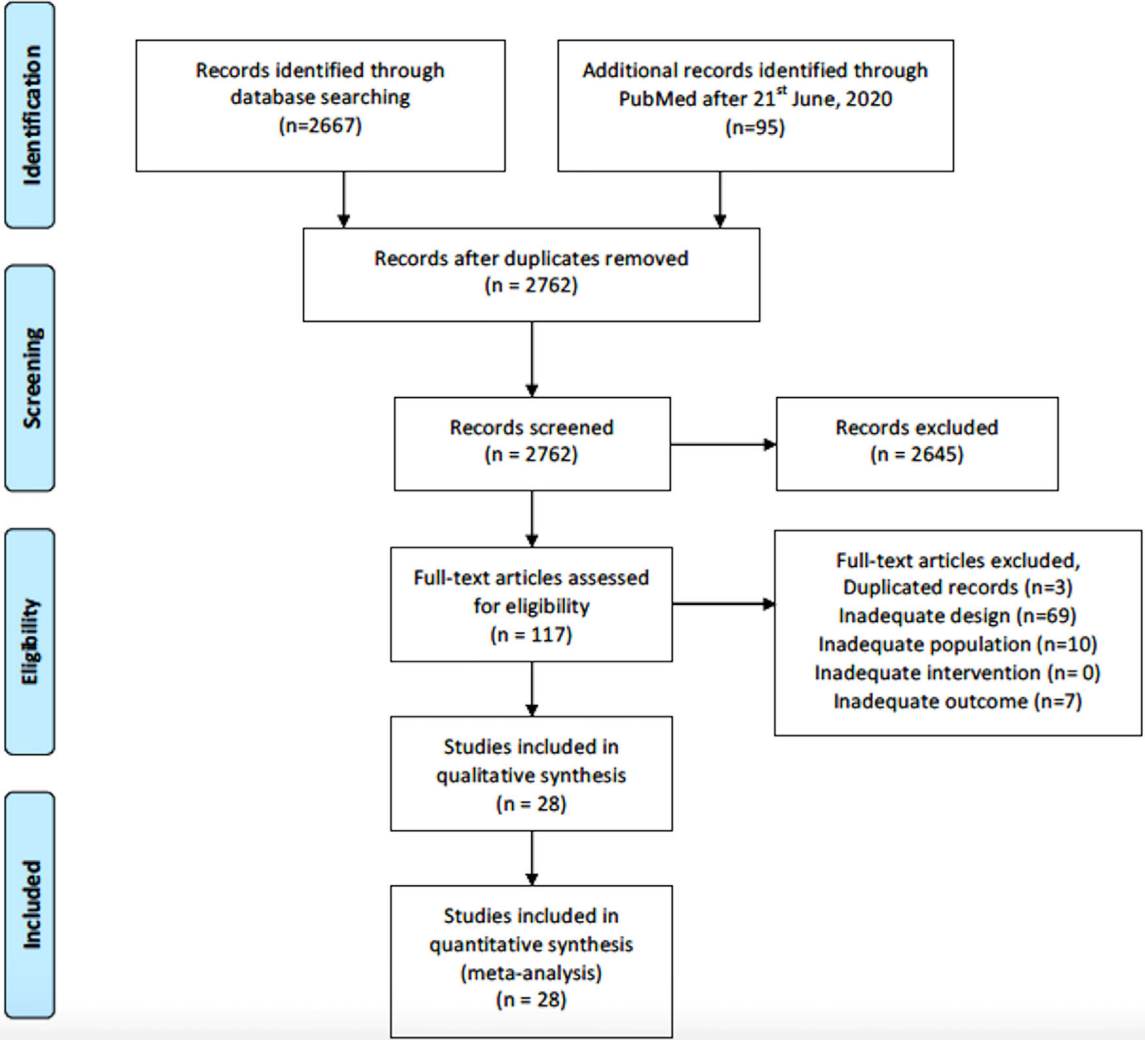
Flow diagram showing the identification of eligible trials and participating trials. A PRISMA flow diagram of the full study‐selection process was shown

Two thousand seven hundred sixty two records were identified and 2654 trials were rejected after title‐abstract screening. Of these, 117 full‐text articles were assessed for eligibility, with 89 studies excluded due to inadequate design, population, or outcome. From June 21, 2020 to November 12, 2020, we updated search results in PubMed and added an additional nine studies to the final analysis (Figure 1).

A total of 28 studies with 73 465 patients were included in this study. There was an equal proportion of males and females, with the age groups primarily from the 60–70 range. The prevalence of hypertension was 12.2–100%, while coronary artery disease (CAD) was 0.4–42.1%. The majority of studies provided data on hospital deaths, and three studies provided mid‐term or long‐term death data. In most studies, groups of patients using either of ACEIs/ARBs were combined for statistical reasons, while a few studies performed separate statistical analyses. The methodological characteristics of the included studies are summarized in Table 1.

**TABLE 1:** The methodological characteristics of the included studies

Time	Study	Country	Type	Total Number	Age^a^	Male/Female^b^	HTN	CAD	AECI/ARB	Non‐AECI/AEB	Mortality
2020.3	Meng 2020^41^	China	Retrospective	417	64/65	24/18	51 (12.23%)	NR	17	25	Event: 0/17,1/25
2020.4	Huang 2020^16^	China	Cohort	50	52.65, 67.77	10/10, 17/13	50 (100%)	NR	20	30	Event: 0/20, 3/30
2020.4	Richardson 2020^8^	USA	Cohort	5700	63	3437/2263	3026 (56.6%)	595 (11.1%)	ACEI 168 ARB 245	953	Event: ACEI vs control 55/168, 254/953 Event: ARB vs control 75/245, 254/953
2020.5	Mehta 2020^42^	USA	Cohort	1735	63, 53	67/49, 792/827	NR	NR	112	570	Event: 8/221, 34/1494
2020.5	Jung 2020^9^	Korea	Cohort	5179	62.5, 41.5	400/362, 1985/2522	1157 (22%)	MI: 49(1%)	762	4417	Crude OR 3.88, Adjusted OR 0.88
2020.6	Bean 2020^43^	UK	Cohort	1200	73.02/65.45	231/168, 455/346	645 (53.8%)	160 (13.3%)	399	801	Crude OR 0.83, Adjusted OR 0.63
2020.6	Zhang 2020^17^	China	Retrospective	1128	64, 64	291/231	1128 (100%)	131 (11.6%)	188	940	HR 0.42, PS‐HR 0.37
2020.6	Otero 2020^11^	Spain	Cohort	965	72.1,56	118/92, 306/499	30.9%	4.4%	213	755	Crude OR 1.49, Adjusted OR 0.62
2020.6	Gao 2020^19^	China	Cohort	710	62.64, 64.84	104/79, 266/261	100%	Angina: 112(15.8%) MI:3(0.4%) PCI/CABG: 37(5.2%)	183	527	Crude HR 0.6, HR 0.8
2020.6	Imam 2020^44^	USA	Cohort	1305	NR	NR	734 (56.2%)	208 (15.9%)	NR	NR	Crude OR 1.55, Adjusted OR 1.2
2020.6	[Peng 2020]^45^	China	Case‐control	112	62(55, 67)	53/59	92 (82.1%)	62 (55.4%)	22	90	Event: 4/17, 18/95
2020.7	Yang 2020^15^	China	Cohort	126	65, 67	21/22, 41/42	126 (100%)	NR	43	83	Event: 2/43, 11/83
2020.7	Fosbol 2020^14^	Demark	Cohort	4480	72.8, 50.1	492/402, 1651/1934	843 (18.8%)	MI: 411(9.2%)	895	3585	HR 0.83
2020.7	Li 2020^20^	China	Cohort	362	65, 67	68/47, 121/126	362 (100%)	62 (17.1%)	115	247	Event: 21/115, 56/247
2020.8	Zhou 2020^40^	China	Cohort	3572	66	1825/1747	NR	NR	906	1812	Event: 70/906, 272/1812 HR 0.39, HTN‐HR 0.32
2020.8	Andrea 2020^18^	Italy	Case series	191	NR	NR	96 (50.3%)	28 (14.7%)	NR	NR	HR 1.8, HTN‐HR 0.5
2020.8	Matsuzawa 2020^46^	Japan	Cohort	151	60±19	13/8, 14/4	39 (25.8%)	MI: 0	21	18	Crude OR 0.53, Adjusted OR 0.36
2020.8	Ran 2020^12^	China	Cohort	803	NR	394/409	803 (100%)	118 (14.7%)	NR	NR	Cure HR 0.75, Adjusted HR 0.08
2020.8	Lee 2020^10^	Korea	Cohort	1609	64.6, 69.0	455/586, 244/324	1609 (100%)	MI: 65(4.0%)	1041	568	Crude OR 0.59, Adjusted OR 0.81
2020.8	Seo 2020^6^	Korea	Case‐control	423	78.4,77.6	73/70,126/145	432 (100%)	MI & stroke: 172′(40.7%)	NR	NR	3‐month of adjusted OR 0.946 6‐month of adjusted OR 0.862 1‐year of adjusted OR 0.875
2020.8	Trifiro 2020^7^	Italy	Cohort	42926	69	2951/1980, 13603/8371	5610 (13.1%	4436 (10.3%)	ARB 897 ACEI 878	1907	ACEI HR 1.12 ARB HR 1.10
2020.8	Felice 2020^47^	Italy	Cohort	133	73.1, 76.2	59/23, 27/24	133 (100%)	56 (42.1%)	82	51	Crude OR 0.41, Adjusted OR 0.56
2020.9	Khan 2020^5^	UK	Cohort	173	66.2, 74.6	20/7, 30/31	88 (50.8%)	NR	27	61	60‐day Event:5/21, 14/61
2020.9	Soleimani 2020^13^	Iran	Cohort	145	68.0, 64.9	72/50, 77/55	NR	NR	122	132	Adjusted OR 2.22, HTN‐ad OR 1.6
2020.9	Pan 2020^48^	China	Cohort	996	70, 69	16/25, 127/114	282 (28.3%)	60 (6.0%)	41	241	Event: 4/41, 63/241
2020.9	Wang 2020^49^	China	Cohort	210	68, 66	38/43, 62/67	210 (100%)	61 (29.0%)	81	129	Event: 7/81, 5/129
2020.9	[Huang 2020]^50^	China	Case‐control	58	64, 64	16/10, 16/16	39 (67.2%)	9 (15.5%)	26	32	Mortality: 7.7%, 3.1%
2020.11	[Zhuang 2020]^51^	China	Case‐control	67	63.4, NR	33/34	67 (100%)	7 (10.4%)	22	45 (included discontinuation group and non‐user)	Event:0,0

*Abbreviations*: ACEI, angiotensin converting enzyme inhibitor; ARB, angiotensin receptor blocker; HTN, hypertension; MI: myocardial infarction; NR, no report; PS, propensity score.

^a^ If the study provided the mean age of two groups, the age was shown separately. If the study did not provide the age of two groups, only the mean age of the whole population was shown.

^b^ If the study provided the male/female number of two groups, the male/female number was shown separately. If the study did not provide the male/female number of two groups, only the male/female number of the whole population was shown.

### 3.2 Risk of bias

Risk of bias assessment was shown in Appendix 2. Three studies were assessed as having serious risk of bias, and 13 studies were found to be moderate risk of bias. Twelve studies were considered as having low risk of bias.

### 3.3 Relationship between ACEIs/ARBs and all‐cause mortality

**FIGURE 2:**
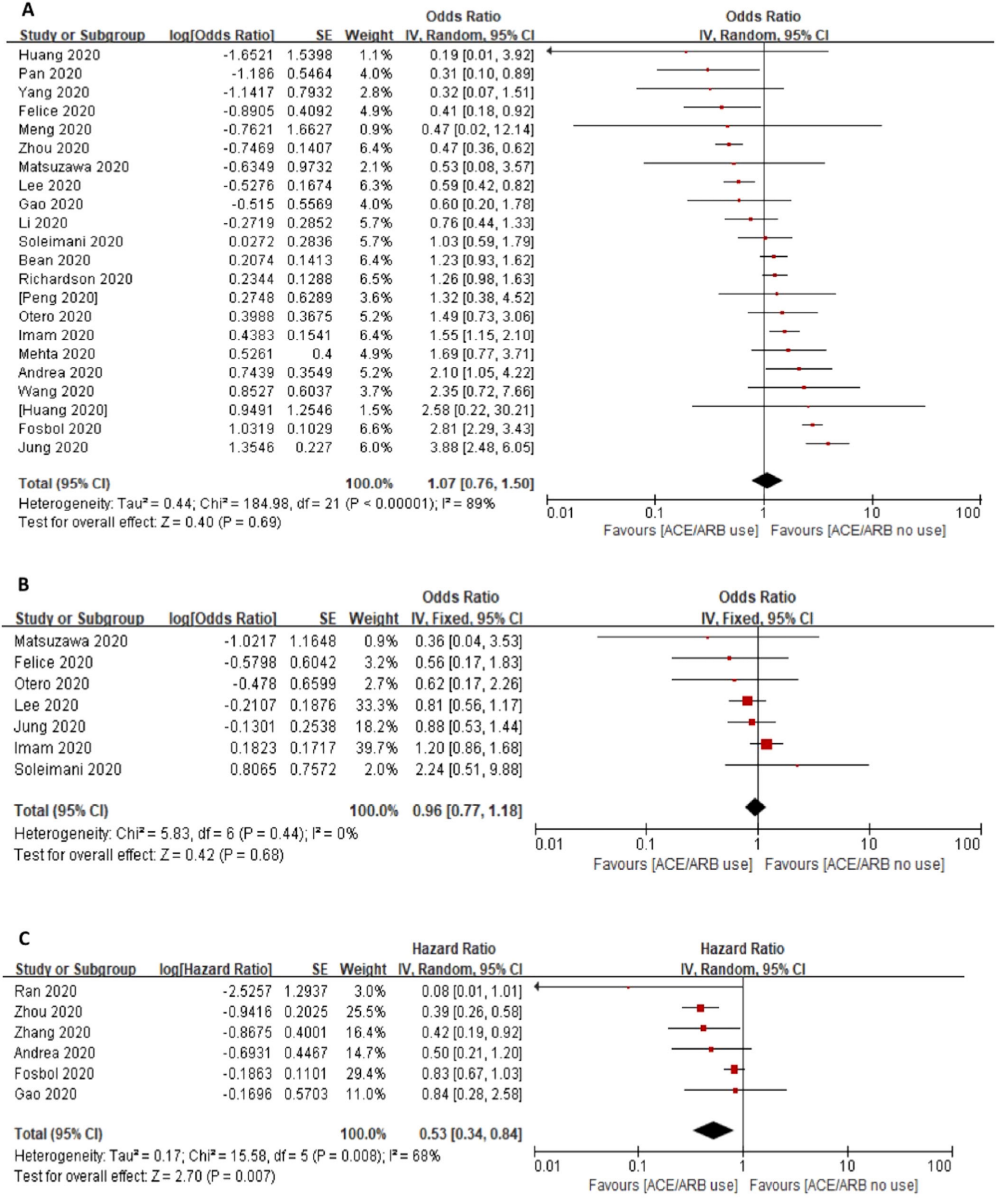
Association between ACEI/ARB use and in‐hospital all‐cause mortality. Pooled risk of in‐hospital all‐cause mortality was shown as crude OR (A), adjusted OR (B), and HR (C) *Abbreviations*: ACEI, angiotensin converting enzyme inhibitor; ARB, angiotensin receptor blocker; HR, hazard ratio; OR, odd ratio.

In patients with COVID‐19, a total of 23 studies with 19,938 patients reported the incidence of all‐cause mortality. Meta‐analysis showed no association between using ACEIs/ARBs and risk of mortality (crude OR = 1.07, 95% CI 0.76–1.50, *p *=.89, *I^2 ^* = 89%). Seven studies with 6260 patients reported adjusted OR and produced a non‐significant association (OR of 0.96, 95% CI 0.77–1.18, *p *= .68, *I^2 ^* = 0%). Six studies with 10 030 patients reported a reduced risk of mortality (HR = 0.53, 95% CI 0.34–0.84, *p *= .007, *I^2 ^* = 68%) (Figure 2).

**FIGURE 3:**
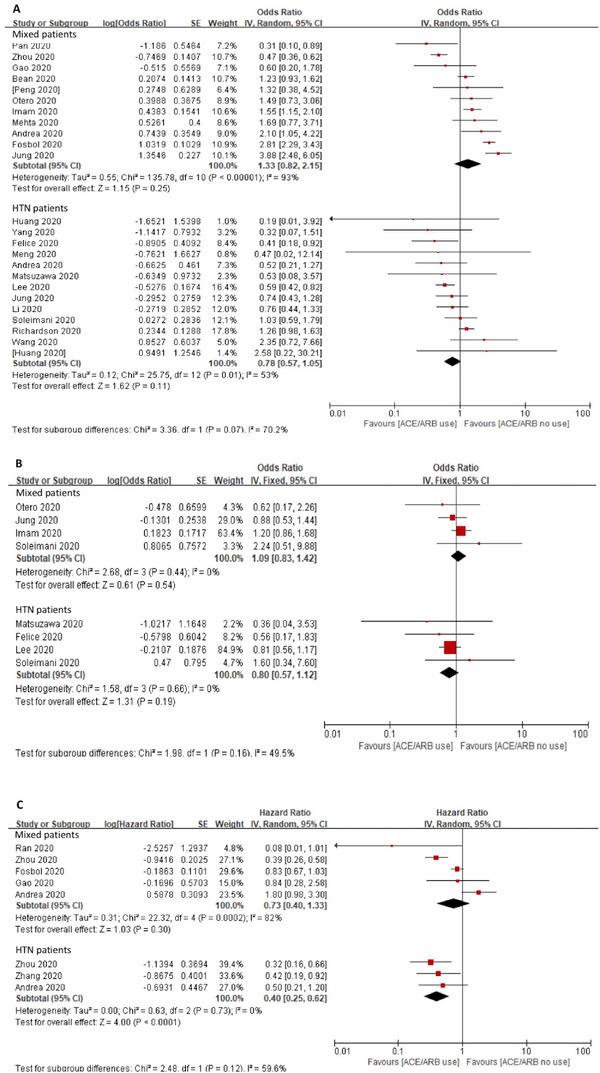
Association between ACEI/ARB use and in‐hospital all‐cause mortality with hypertension subgroup. Pooled risk of in‐hospital all‐cause mortality was shown as crude OR (A), adjusted OR (B), and HR (C). Population were divided into mix population and hypertensive population. Some studies provided the effect size of overall population and hypertension subgroup. The effect size was pooled separately. *Abbreviations*: ACEI, angiotensin converting enzyme inhibitor; ARB, angiotensin receptor blocker; HR, hazard ratio; OR, odd ratio.

In the hypertension subgroup (Appendix 3, Figure 10–13), 13 studies with 4887 patients reported a crude OR of mortality (OR of 0.78, 95% CI 0.57–1.05, *p *= .11, *I* ^2 ^= 53%). Four studies reported adjusted OR with 1927 patients produced a non‐significant (OR of 0.80, 95% CI 0.57–1.12, *p *= .19, *I* ^2^*^^* = 0%) (Figure 3). Three studies reported HR found a lower risk of mortality (HR = 0.40, 95% CI 0.25–0.62, *p *< .0001, *I^2 ^* = 0%).

### 3.4 Relationship between ACEIs/ARBs and other clinical outcomes

Three studies provided long‐term mortality data, but the results could not be combined. Khan^5^ reported a 60‐day mortality event for the ACEIs group versus no use group (5/27 versus 14/61, respectively). Seo and Son^6^ provided 3‐month, 6‐month, and 1‐year mortality with an adjusted OR of 0.946 (95% CI 0.59–1.517), 0.862 (95% CI 0.54–1.375), 0.875 (95% CI 0.548–1.396), respectively. Trifiro and coworker^7^ provided 3‐month mortality HR for ACEIs (1.10, 95% CI 1.03–1.17) and ARBs (1.12, 95% CI 1.05–1.20).

Seven studies with 4,576 patients reported the risk of ventilation and the result showed no association (crude OR of 1.29, 95% CI 0.85–1.96, *p *= .24, *I* ^2^*^^* = 59%, Appendix 3, Figure 5). Among them five studies with 2,572 patients reported an adjusted *OR* for ventilation, still without association with ACEIs/ARBs use (adjusted OR = 1.07, 95% CI 0.74–1.54, *p *= .72, *I* ^2^*^^* = 0%, Appendix 3, Figure 6). We narratively synthesized the results of duration of hospital stay, but did not show a significant difference. Only Richardson and coworker^8^ reported readmission data, and again, there was not significantly different between the ACEIs group (3/168) and the ARBs group (3/245). Five studies provided the data of heart failure (HF). The HF event in Jung and coworker^9^ was reported for the hypertension subgroup. Only two studies^10^, ^11^ with 2574 patients reported a crude OR of 1.28 (95% CI 0.49–3.35, *p *= .62, *I^2 ^* = 83%, Appendix 3, Figure 7), and an adjusted OR 1.34 (95% CI 0.39–4.77, *p *= .20, *I^2 ^* = 0%, Appendix 3, Figure 8), respectively. Ran and coworker^12^ reported HR for HF of 1.39 (95% CI 0.38–5.02). Soleimani and coworker^13^ reported data for cardiac injury. The adjusted OR was 2.17 (95% CI 0.57–8.32). In the hypertension subgroup, the adjusted OR was 1.91 (95% CI 0.52–7.07).

### 3.5 Subgroup analysis

We preset the subgroup analysis for sex and age in the prior subgroup. However, only few individual studies were available. Soleimani and coworker^13^ provided the adjusted OR (for men, OR of 1.18, 95% CI 0.45–3.10, and for women, OR of 0.58, 95% CI 0.17–1.95). While Fosbøl and coworker^14^ provided adjusted HR (for men, HR of 0.81, 95% CI 0.58–1.15 and for women, HR of 0.81, 95% CI 0.62–1.08). Generally, patients in the ACEIs/ARBs group were older than those in the control group, with the age difference ranging from 8 to 22 years (Appendix 3). But no study carried out comparison between groups aged ≥65 years and aged < 65 years. Therefore, we made a posterior subgroup analysis based on the *p*‐values of age variable in the original individual study, for example, ``*p *> .05,’’ ``*p* ≤ .05,’’ and ``absent *p* value’’ with the crude ORs of 0.78 (95% CI 0.51–1.19, *p *= .24, *I^2 ^* = 28%), 1.30 (95% CI 0.74–2.30, *p *= .36, *I^2 ^* = 89%), and 1.28 (95% CI 0.64–2.56, *p *= .49, *I^2 ^* = 96%), respectively (Appendix 3, Figure 13).

**FIGURE 4:**
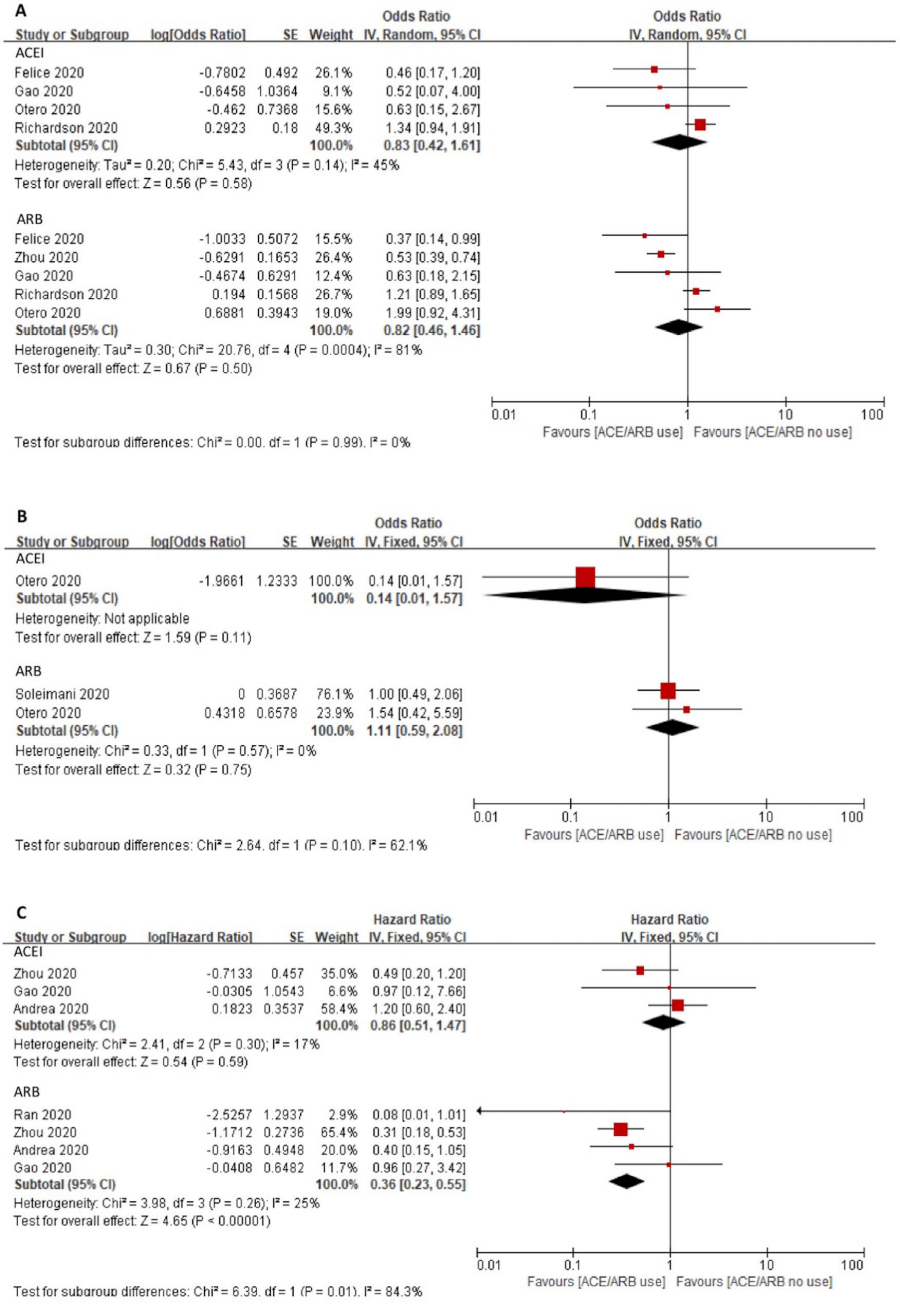
Association between ACEI or ARB use alone and in‐hospital all‐cause mortality. Pooled risk of in‐hospital all‐cause mortality was shown as crude OR (A), adjusted OR (B), and HR (C). Studies were divided into ACEI subgroup and ARB subgroup. The effect size was pooled separately *Abbreviations*: ACEI, angiotensin converting enzyme inhibitor; ARB, angiotensin receptor blocker; HR, hazard ratio; OR, odd ratio.

Analysis of the ACEIs and ARBs use as separate subgroups is shown in Figure 4. For the ACEIs group, four studies (one study did not provide sample size, and the other three *n* = 1791) yielded a crude OR of 0.83 (95% CI 0.42–1.61, *p *= .58, *I^2 ^* = 45%). Only one study showed an adjusted *OR* 0.14 (95% CI 0.01–1.57, *p *= .11). Three studies with 3234 patients provided a HR of 0.86 (95% CI 0.51–1.47, *p *= .59, *I^2 ^* = 17%). None of the results showed a significant difference between the groups. In the ARBs subgroup, five studies (one study did not provide sample size, and the other four studies had *n* = 4797) showed a crude OR of 0.82 (95% CI 0.46–1.46, *p *= .50, *I* ^2^*^^* = 81%). Two studies (without sample size) had an adjusted OR of 1.11 (95% CI 0.59–2.08, *p *= .75, *I^2 ^* = 0%). Four studies with 4357 patients indicated a lower risk of mortality in ARBs group (HR = 0.36, 95% CI 0.21–0.56, *p *< .0001, *I^2 ^* = 25%, Figure 4) compared with non‐user. Three studies made head to head comparison between ACEIs and ARBs, but showed no significant association (crude OR of 1.11, 95% CI 0.75–1.64, *p *= .60, *I* ^2^*^^* = 0%, Appendix 3, Figure 19).

### 3.6 Sensitivity analysis

Additionally, three studies^5^, ^15^, ^16^ with serious risk of bias were excluded from the analyses of crude OR, adjusted OR for all‐cause mortality, and we still did not find significant associations (crude OR of 1.13, 95% CI 0.80–1.60, *p *= .48, *I^2 ^* = 89%, and adjusted OR of 0.96, 95% CI 0.77–1.18, *p *= .68, *I* ^2^*^^* = 0%, respectively. While for *HR*, it is still significantly favored ACEIs/ARBs use (HR = 0.53, 95% CI 0.34–0.84, *p *= .007, *I^2 ^* = 68%). In the hypertension subgroup, the crude *OR*, adjusted *OR* showed non‐significant association (crude OR of 0.83, 95% CI 0.60–1.16, *p *= .27, *I* ^2^*^^* = 60%, and adjusted OR of 0.80, 95% CI 0.57–1.12, *p *= .19, *I^2 ^* = 0%, Figure 3). For HR again, the results favored ACEIs/ARBs use (HR of 0.40, 95% CI 0.25–0.62, *p *< .0001, *I^2 ^* = 0%) (Appendix 3).

## 4 DISCUSSION

The inconsistent conclusions about the use of ACEIs/ARBs call for more rigorous studies to demonstrate the effect of ACEIs/ARBs in different population. Several relevant systematic reviews and meta‐analysis summarized available evidence and suggested continue use of ACEIs/ARBs based on the lack of association of the treatment on the risk of mortality or severity of outcomes. By using strict paper inclusion criteria, and adjusting for confounding effect whenever possible, our systematic review provided relatively higher quality of evidence to increase the assurance of using ACEIs or ARBs on COVID‐19 patients. We identified 28 studies with 73 465 COVID‐19 patients and confirmed a non‐significant association between ACEIs/ARBs use and the risk of all‐cause mortality. Besides, our study found ACEIs/ARBs reduced the risk of 30‐day all‐cause mortality in both the entire population and the hypertension subgroup. Since SARS‐Cov‐2 can bind ACE2 and allows the virus to invade target cells, which may lead to activate immune response and cause lung injury, there still have discussion about using ARBs to replace ACEIs according to this mechanism. Our head to head comparison of ACEIs versus ARBs showed no difference in association with the risk of mortality. Furthermore, our study showed that ACEIs/ARBs use was not associated with other not fully addressed secondary outcomes such as long‐term mortality, ventilation, readmission, and cardiac adverse events.

At present, there are more than 10 published meta‐analyses^21^, ^22^, ^23^, ^24^, ^25^, ^26^, ^27^, ^28^, ^29^, ^30^, ^31^, ^32^, ^33^, ^34^, ^35^, ^36^, ^37^, ^38^ to reveal the association of using ACEIs/ARBs with the risk of adverse outcomes in COVID‐19 patients. Unlike previous systematic review and meta‐analyses, we reduced potential bias by excluding unpublished studies since they did not undergo peer‐review process and their conclusions may subject to uncertainty. Also, we limited the study to those with the patients using PCR or genetic test to confirm COVID‐19 infection due to the consideration that clinical suspicion of COVID‐19 may hard to be distinguished from other cause of pneumonia. We use ROBINS‐I to evaluate the risk of bias in the body of evidence and found a moderate to low risk of bias. After excluded three studies with serious bias, the direction of association did not change (Appendix3, figure 20–27).

High heterogeneity was present in pooled crude OR. Some studies^7^, ^17^, ^18^, ^19^, ^20^ indicated that age and comorbidities such as hypertension, CAD, HF were significantly associated with mortality in patients with COVID‐19. In the whole population, some results showed favoring use of ACEIs/ARBs^23,37,39^ to reduce the risk of mortality, while more studies showed non‐significant associations between them.^21^ ^,24,^ ^27,28,30,35^ Among hypertensive patients, more studies supported lower risk of mortality in ACEIs/ARBs group,^21^, ^23^, ^29^, ^31^, ^37^ while fewer studies^26^, ^30^ did not support. In the Hasan study,^39^ the adjusted OR and HR significantly favored using ACEIs/ARBs for reducing the risk of death. However, significance was lost when death and serious diseases were taken as joint endpoints. The heterogeneity may be explained by confounding effect of age, serious of diseases and comorbidities. So, we calculated the adjusted OR and HR separately and found that ACEIs/ARBs use was not associated with in‐hospital all‐cause mortality. These results are consistent with previous study and stop using is not recommended. In our study, when the risk was evaluated by HR, we found using ACEIs/ARBs was associated with a decreased risk of 30‐day all‐cause mortality. Similar results are found in the hypertensive subgroup. This result indicated that time effect should be considered when evaluate the clinical outcomes of using ACEIs/ARBs.

Our study conducted some subgroup analysis for the association of adverse outcomes and the use of ACEIs/ARBs according to our protocol. Hypertension was the most common comorbidities and ACEIs/ARBs user population. Our study showed that adjusted *OR* was not significantly favor ACEIs/ARBs use, but *HR* showed an opposite result. In Ren 2020′s review, they observed a significant reduced severity of COVID‐19 infection and risk of mortality (measured by *OR*) and they suggested favor use of ACEIs/ARBs in hypertension groups. Since the data did not provide comparison between age group (≥65‐years‐old versus < 65‐years‐old), our study made three subgroup analyses according to the statistical difference of age variable in the original studies, for example, ‘*p *> .05′, ‘*p*≤.05′, and ``absent *p* value’’ (Appendix 3 Figure 13), and produced crude ORs of 0.78 (95% CI 0.51–1.19), 1.30 (95% CI 0.74–2.30) and 1.28 (95% CI 0.64–2.56), respectively. Interestedly, from the data with *p *> .05 for age, it tends to favor ACEIs/ARBs use. So, age maybe an important factor causing heterogeneity and changing the direction of the association between ACEIs/ARBs and risk of mortality. However, longer term follow‐up study by Tirifiro 2020′s^7^ showed that ACEIs/ARBs use had the same effect as other antihypertensive drugs did. When comparing the effects of ACEIs versus ARBs, the available data showed that ARBs reduced the risk of mortality. But the result is still controversial.

Since data are limited, we did not pool the studies about continue using and stop using ACEIs/ARBs after COVID‐19 infection. The study by Soleimani 2020 suggested that the death rate in patients who stopped using ACEIs/ARBs was higher than that of other groups. On the other side, a trail (NCT04338009) conducted in the USA comparing the mortality rate between discontinuation versus continuation using ACEIs/ARBs found that the mortality was 12.99% versus 14.67%, which suggested favor discontinuous use of ACEIs/ARBs. However, the reason for interrupting use of ACEIs/ARBs was not well explained.

There are some limitations in this study. First, the original observational studies inherit high risk of bias and reduce the quality level of evidence. So using strict paper inclusion criteria, and adjusting for confounding effect whenever possible, we got our data have a moderate to low risk of bias (Appendix 2). Second, although we used adjusted OR and adjusted HR whenever possible to reduce the confounding effect, some bias still cannot be fully addressed. For example, patients with hypertension and other cardiovascular disease needed to be prescribed ACEIs or ARBs may have different disease risk and prognosis even though they did not have COVID‐19. Due to the limitation of data, this meta‐analysis cannot control the bias caused by different cardiovascular comorbidities. Third, although our study found using ACEIs/ARBs statistically significant reduced the risk of mortality evaluated by HR, it should be noticed that among the six studies reporting HR, four were conducted in Wuhan, China, and two were in Europe, which may influence the representativeness of population.

## 5 CONCLUSION

Based on the available data, we conclude that using ACEIs/ARBs is not associated with the risk of in‐hospital all‐cause mortality in COVID‐19 patients, but may be associated with a decreased risk of 30‐day all‐cause mortality. Patients with hypertension may benefit from using ACEIs/ARBs.

## FUNDING

Disciplines Construction Project of Peking Union Medical College (No.201920202102) initiated the study. CAMS Innovation Fund for Medical Science (No. 2018‐I2M‐1‐002) supported data extraction and the assessment of the risk of bias.

## CONFLICT OF INTERESTS

There are no conflicts of interest to declare.

## AUTHOR CONTRIBUTION

Deping Liu and Zeng Ping conducted this study. Deping Liu, Ping Zeng, Na Jia, Yan Wang, Sai Zhao, Jun Xia developed the protocol. Na Jia, Xuelin Sun, Yan Wang screened the literatures. Ping Zeng made the final decision if there is disagreement. Wenjie Chi, Sitong Dong extracted the data. Sitong Dong and Sai Zhao did the risk of bias assessment. Guifang Zhang did additional search and the data analysis. Na Jia drafted the manuscript. Ping Zeng, Deping Liu, Sai Zhao, Sitong Dong revised the manuscript.

## Supporting information

Supporting material

Supporting material

Supporting material

## References

[1] Bosch BJ , van der Zee R , de Haan CA , et al. The coronavirus spike protein is a class I virus fusion protein: structural and functional characterization of the fusion core complex. J. Virol. 2003;77(16):8801‐8811. published Online First: 2003/07/30. 12885899 10.1128/JVI.77.16.8801-8811.2003 PMC167208

[2] Shang J , Ye G , Shi K , et al. Structural basis of receptor recognition by SARS‐CoV‐2. Nature. 2020;581(7807):221‐224. published Online First: 2020/04/01. 32225175 10.1038/s41586-020-2179-y PMC7328981

[3] Bozkurt B , Kovacs R , Harrington B . Joint HFSA/ACC/AHA statement addresses concerns Re: using RAAS Antagonists in COVID‐19. J. Cardiac Fail. 2020;26(5):370. published Online First: 2020/05/23. 10.1016/j.cardfail.2020.04.013 PMC7234783 32439095

[4] Cardiology CoHotESo . Position statement of the ESC Council on Hypertension on ACE‐inhibitors and angiotensin receptor blockers 2020. https://www.escardio.org/Councils/Council‐on‐Hypertension‐(CHT)/News/position‐statement‐of‐the‐esc‐council‐on‐hypertension‐on‐ace‐inhibitors‐and‐ang

[5] Khan KS , Reed‐Embleton H , Lewis J , et al. Angiotensin converting enzyme inhibitors do not increase the risk of poor outcomes in COVID‐19 disease. A multi‐centre observational study. Scot Med. J. 2020;65(4):149‐153. published Online First: 2020/09/03. 32873147 10.1177/0036933020951926 PMC7468667

[6] Seo J , Son M . Update on association between exposure to renin‐angiotensin‐aldosterone system inhibitors and coronavirus disease 2019 in South Korea. Korean J. Internal Med. 2020. 2020:380. 10.3904/kjim. published Online First: 2020/09/03. PMC8009148 32872736

[7] Trifirò G , Massari M , Da Cas R , et al. Renin‐angiotensin‐aldosterone system inhibitors and risk of death in patients hospitalised with COVID‐19: a retrospective Italian Cohort study of 43,000 patients. Drug Saf. 2020;43(12):1297‐1308. published Online First: 2020/08/28. 32852721 10.1007/s40264-020-00994-5 PMC7450482

[8] Richardson S , Hirsch JS , Narasimhan M , et al. Presenting characteristics, comorbidities, and outcomes among 5700 patients hospitalized with COVID‐19 in the New York City Area. JAMA. 2020;323(20):2052‐2059. published Online First: 2020/04/23. 32320003 10.1001/jama.2020.6775 PMC7177629

[9] Jung SY , Choi JC , You SH , et al. Association of Renin‐angiotensin‐aldosterone System Inhibitors With Coronavirus Disease 2019 (COVID‐19)‐ Related Outcomes in Korea: a Nationwide Population‐based Cohort Study. Clin. Infect. Dis. 2020;71(16):2121‐2128. published Online First: 2020/05/23. 32442285 10.1093/cid/ciaa624 PMC7314113

[10] Lee J , Jo SJ , Cho Y , et al. Effects of renin‐angiotensin system blockers on the risk and outcomes of severe acute respiratory syndrome coronavirus 2 infection in patients with hypertension. Korean J. Int. Med. 2021. published Online First: 2020/09/03. 10.3904/kjim.2020.390 PMC8009159 32872731

[11] López‐Otero D , López‐Pais J , Cacho‐Antonio CE , et al. Impact of angiotensin‐converting enzyme inhibitors and angiotensin receptor blockers on COVID‐19 in a western population. CARDIOVID registry. Revista espanola de cardiologia (English ed). 2021;74(2):175‐182. published Online First: 2020/07/01. 32600991 10.1016/j.rec.2020.05.018 PMC7274611

[12] Ran J , Song Y , Zhuang Z , et al. . Blood pressure control and adverse outcomes of COVID‐19 infection in patients with concomitant hypertension in Wuhan, China. Hypertens. Res. 2020;43(11):1267‐1276. published Online First: 2020/08/29. 32855527 10.1038/s41440-020-00541-w PMC7450040

[13] Soleimani A , Kazemian S , Karbalai Saleh S , et al. Effects of angiotensin receptor blockers (ARBs) on In‐Hospital Outcomes of Patients With Hypertension and Confirmed or Clinically Suspected COVID‐19. Am. J. Hypertens. 2020;33(12):1102‐1111. published Online First: 2020/09/14. 32920644 10.1093/ajh/hpaa149 PMC7543264

[14] Fosbøl EL , Butt JH , Østergaard L , et al. Association of Angiotensin‐Converting Enzyme Inhibitor or Angiotensin Receptor Blocker Use With COVID‐19 Diagnosis and Mortality. JAMA. 2020;324(2):168‐177. published Online First: 2020/06/20. 32558877 10.1001/jama.2020.11301 PMC7305566

[15] Yang G , Tan Z , Zhou L , et al. Effects of Angiotensin II Receptor Blockers and ACE (Angiotensin‐Converting Enzyme) Inhibitors on Virus Infection, Inflammatory Status, and Clinical Outcomes in Patients With COVID‐19 and Hypertension: a Single‐Center Retrospective Study. Hypertension. 2020;76(1):51‐58. published Online First: 2020/04/30. 32348166 10.1161/HYPERTENSIONAHA.120.15143

[16] Huang Z , Cao J , Yao Y , et al. The effect of RAS blockers on the clinical characteristics of COVID‐19 patients with hypertension. Ann. Trans. Med. 2020;8(7):430. published Online First: 2020/05/13. 10.21037/atm.2020.03.229 PMC7210199 32395474

[17] Zhang P , Zhu L , Cai J , et al. Association of Inpatient Use of Angiotensin Converting Enzyme Inhibitors and Angiotensin II Receptor Blockers with Mortality Among Patients With Hypertension Hospitalized With COVID‐19. Circ. Res. 2020. 10.1161/CIRCRESAHA.120.317134 PMC7265882 32302265

[18] Conversano A , Melillo F , Napolano A , et al. Renin‐Angiotensin‐Aldosterone System Inhibitors and Outcome in Patients With SARS‐CoV‐2 Pneumonia: a Case Series Study. Hypertension. 2020;76(2):e10‐e12. published Online First: 2020/05/10. 32383626 10.1161/HYPERTENSIONAHA.120.15312

[19] Gao C , Cai Y , Zhang K , et al. Association of hypertension and antihypertensive treatment with COVID‐19 mortality: a retrospective observational study. European heart journal. 2020;41(22):2058‐2066. published Online First: 2020/06/05. 32498076 10.1093/eurheartj/ehaa433 PMC7314067

[20] Li J , Wang X , Chen J , et al. Association of Renin‐Angiotensin System Inhibitors With Severity or Risk of Death in Patients With Hypertension Hospitalized for Coronavirus Disease 2019 (COVID‐19) Infection in Wuhan, China. JAMA Cardiol. 2020;5(7):825‐830. published Online First: 2020/04/24. 32324209 10.1001/jamacardio.2020.1624 PMC7180726

[21] Ren L , Yu S , Xu W , et al. Lack of association of antihypertensive drugs with the risk and severity of COVID‐19: a meta‐analysis. J Cardiol. 2020. 10.1016/j.jjcc.2020.10.015. published Online First: 2020/11/11. PMC7605745 33168337

[22] Pranata R , Permana H , Huang I , et al. The use of renin angiotensin system inhibitor on mortality in patients with coronavirus disease 2019 (COVID‐19): a systematic review and meta‐analysis. Diabetes & metabolic syndrome. 2020;14(5):983‐990. published Online First: 2020/07/03. 32615377 10.1016/j.dsx.2020.06.047 PMC7319940

[23] Baral R , White M , Vassiliou VS . Effect of Renin‐Angiotensin‐Aldosterone System Inhibitors in Patients with COVID‐19: a Systematic Review and Meta‐analysis of 28,872 Patients. Current atherosclerosis reports. 2020;22(10):61. published Online First: 2020/08/25. 32830286 10.1007/s11883-020-00880-6 PMC7443394

[24] Zhang X , Yu J , Pan LY , et al. ACEIs/ARB use and risk of infection or severity or mortality of COVID‐19: a systematic review and meta‐analysis. Pharmacological research. 2020;158:104927. published Online First: 2020/05/19. 32422341 10.1016/j.phrs.2020.104927 PMC7227582

[25] Pirola CJ , Sookoian S . Estimation of Renin‐Angiotensin‐Aldosterone‐System (RAAS)‐Inhibitor effect on COVID‐19 outcome: a Meta‐analysis. The Journal of infection. 2020;81(2):276‐281. published Online First: 2020/06/01. 32474043 10.1016/j.jinf.2020.05.052 PMC7255761

[26] Usman MS , Siddiqi TJ , Khan MS , et al. A Meta‐analysis of the Relationship Between Renin‐Angiotensin‐Aldosterone System Inhibitors and COVID‐19. The American journal of cardiology. 2020;130:159‐161. published Online First: 2020/07/07. 32624189 10.1016/j.amjcard.2020.05.038 PMC7266568

[27] Patoulias D , Katsimardou A , Stavropoulos K , et al. Renin‐Angiotensin System Inhibitors and COVID‐19: a Systematic Review and Meta‐Analysis. Evidence for Significant Geographical Disparities. Current hypertension reports. 2020;22(11):90. published Online First: 2020/09/11. 32910274 10.1007/s11906-020-01101-w PMC7481766

[28] Greco A , Buccheri S , D'Arrigo P , et al. Outcomes of renin‐angiotensin‐aldosterone system blockers in patients with COVID‐19: a systematic review and meta‐analysis. European heart journal Cardiovascular pharmacotherapy. 2020;6(5):335‐337. published Online First: 2020/07/17. 32671399 10.1093/ehjcvp/pvaa074 PMC7454531

[29] Guo X , Zhu Y , Hong Y . Decreased Mortality of COVID‐19 With Renin‐Angiotensin‐Aldosterone System Inhibitors Therapy in Patients With Hypertension: a Meta‐Analysis. Hypertension. 2020;76(2):e13‐e14. published Online First: 2020/05/28. 32458694 10.1161/HYPERTENSIONAHA.120.15572

[30] Flacco ME , Acuti Martellucci C , Bravi F , et al. Treatment with ACE inhibitors or ARBs and risk of severe/lethal COVID‐19: a meta‐analysis. Heart (British Cardiac Society). 2020;106(19):1519‐1524. published Online First: 2020/07/03. 32611676 10.1136/heartjnl-2020-317336 PMC7371482

[31] Ssentongo AE , Ssentongo P , Heilbrunn ES , et al. Renin‐angiotensin‐aldosterone system inhibitors and the risk of mortality in patients with hypertension hospitalised for COVID‐19: systematic review and meta‐analysis. Open Heart. 2020;7(2). published Online First: 2020/11/07. 10.1136/openhrt-2020-001353 PMC7646321 33154144

[32] Chan CK , Huang YS , Liao HW , et al. Renin‐Angiotensin‐Aldosterone System Inhibitors and Risks of Severe Acute Respiratory Syndrome Coronavirus 2 Infection: a Systematic Review and Meta‐Analysis. Hypertension. 2020;76(5):1563‐1571. published Online First: 2020/09/02. 32869673 10.1161/HYPERTENSIONAHA.120.15989 PMC7485525

[33] Barochiner J , Martínez R . Use of inhibitors of the renin‐angiotensin system in hypertensive patients and COVID‐19 severity: a systematic review and meta‐analysis. J. Clin. Pharm. Ther. 2020;45(6):1244‐1252. published Online First: 2020/08/09. 32767823 10.1111/jcpt.13246 PMC7436708

[34] Koshy AN , Murphy AC , Farouque O , et al. Renin‐angiotensin system inhibition and risk of infection and mortality in COVID‐19: a systematic review and meta‐analysis. Intern. Med. J. 2020;50(12):1468‐1474. published Online First: 2020/11/17. 33191600 10.1111/imj.15002 PMC7753674

[35] Lo KB , Bhargav R , Salacup G , et al. Angiotensin converting enzyme inhibitors and angiotensin II receptor blockers and outcomes in patients with COVID‐19: a systematic review and meta‐analysis. Expert Rev. Cardiovasc. Ther. 2020;18(12):919‐930. published Online First: 2020/09/19. 32945216 10.1080/14779072.2020.1826308

[36] Xue Y , Sun S , Cai J , et al. Effects of ACEI and ARB on COVID‐19 patients: a meta‐analysis. JRAAS. 2020;21(4):1470320320981321. published Online First: 2020/12/17. 33325306 10.1177/1470320320981321 PMC7747108

[37] Liu X , Long C , Xiong Q , et al. Association of angiotensin converting enzyme inhibitors and angiotensin II receptor blockers with risk of COVID‐19, inflammation level, severity, and death in patients with COVID‐19: a rapid systematic review and meta‐analysis. Clin Cardiol. 2020. published Online First: 2020/08/07. 10.1002/clc.23421 PMC7436520 32757246

[38] Kurdi A , Abutheraa N , Akil L , et al. A systematic review and meta‐analysis of the use of renin‐angiotensin system drugs and COVID‐19 clinical outcomes: what is the evidence so far?. Pharmacol. Res. Perspect. 2020;8(6):e00666. published Online First: 2020/10/22. 33084232 10.1002/prp2.666 PMC7575889

[39] Hasan SS , Kow CS , Hadi MA , et al. Mortality and Disease Severity Among COVID‐19 Patients Receiving Renin‐Angiotensin System Inhibitors: a Systematic Review and Meta‐analysis. Am. J. Cardiovasc. Drugs. 2020;20(6):571‐590. published Online First: 2020/09/13. 32918209 10.1007/s40256-020-00439-5 PMC7486167

[40] Zhou F , Liu YM , Xie J , et al. Comparative impacts of angiotensin converting enzyme inhibitors versus angiotensin II receptor blockers on the risk of COVID‐19 mortality. Hypertension. 2020. published Online First: 06/05. 10.1161/HYPERTENSIONAHA.120.15622 32493070

[41] Meng J , Xiao G , Zhang J , et al. Renin‐angiotensin system inhibitors improve the clinical outcomes of COVID‐19 patients with hypertension. Emerging Microbes Infect. 2020;9(1):757‐760. published Online First: 04/02. 10.1080/22221751.2020.1746200 PMC7170368 32228222

[42] Mehta N , Mazer‐Amirshahi M , Alkindi N , et al. Pharmacotherapy in COVID‐19; A narrative review for emergency providers. Am. J. Emerg. Med. 2020. published Online First: 04/28. 10.1016/j.ajem.2020.04.035 PMC7158837 32336586

[43] Bean DM , Kraljevic Z , Searle T , et al. ACE‐inhibitors and Angiotensin‐2 Receptor Blockers are not associated with severe SARS‐COVID19 infection in a multi‐site UK acute Hospital Trust. Eur. J. Heart Fail. 2020. 10.1002/ejhf.1924 PMC7301045 32485082

[44] Imam Z , Odish F , Gill I , et al. Older age and comorbidity are independent mortality predictors in a large cohort of 1305 COVID‐19 patients in Michigan, United States. J. Internal Med. 2020;288(4):469‐476. published Online First: 2020/06/05. 32498135 10.1111/joim.13119 PMC7300881

[45] Peng Y , Meng K , Guan H , et al. Clinical characteristics and outcomes of 112 cardiovascular disease patients infected by 2019‑nCoV]. Chin. J. Cardiol. 2020;6(48):450‐455. 10.3760/cma.j.cn112148-20200220-00105 32120458

[46] Matsuzawa Y , Ogawa H , Kimura K , et al. Renin‐angiotensin system inhibitors and the severity of coronavirus disease 2019 in Kanagawa, Japan: a retrospective cohort study. Hypertens. Res. 2020;43(11):1257‐1266. published Online First: 2020/08/21. 32820236 10.1038/s41440-020-00535-8

[47] Felice C , Nardin C , Di Tanna GL , et al. Use of RAAS Inhibitors and Risk of Clinical Deterioration in COVID‐19: results From an Italian Cohort of 133 Hypertensives. Am. J. Hypertens. 2020;33(10):944‐948. published Online First: 2020/06/09. 32511678 10.1093/ajh/hpaa096 PMC7314218

[48] Pan W , Zhang J , Wang M , et al. Clinical Features of COVID‐19 in Patients With Essential Hypertension and the Impacts of Renin‐angiotensin‐aldosterone System Inhibitors on the Prognosis of COVID‐19 Patients. Hypertension. 2020;76(3):732‐741. published Online First: 2020/07/14. 32654555 10.1161/HYPERTENSIONAHA.120.15289

[49] Wang Z , Zhang D , Wang S , et al. A Retrospective Study from 2 Centers in China on the Effects of Continued Use of Angiotensin‐Converting Enzyme Inhibitors and Angiotensin II Receptor Blockers in Patients with Hypertension and COVID‐19. Med. Sci. Monit. 2020;26:e926651. published Online First: 2020/09/25. 32969367 10.12659/MSM.926651 PMC7523417

[50] Huang W , Li T , Ling Y , et al. [Effects of angiotensin converting enzyme inhibitor /angiotensin receptor blocker on clinical characteristics of coronavirus disease 2019 patients with hypertension]. Chin. J. Intern. Med. 2020;9(59):689‐694. 10.3760/cma.j.cn112138-20200229-00155 32838499

[51] Zhuang X , Wang W , Zhao X , et al. [Effect of angiotensin‐converting enzyme inhibitor and angiotensin receptor blocker on the outcome of hospitalization of patients with hypertension and novel coronavirus pneumonia]. Chin. J. Hypertens. 2020;28(11):37‐44.

