# Supplementary material for: Influence of angiotensin converting enzyme inhibitors/angiotensin receptor blockers on the risk of all‐cause mortality and other clinical outcomes in patients with confirmed COVID‐19: A systemic review and meta‐analysis
Source: J Clin Hypertens (Greenwich). 2021 Jul 28;23(9):1651–63. doi: 10.1111/jch.14329 (PMC8420264; doi:10.1111/jch.14329)
Supplement: Supplementary file 1 — Supporting material [file JCH-23-1651-s003.docx]

**Search Report** – SRS200228 update from March to June 2020

**Clinical question**: Does the use of ACE/ARB increase the risk of mortality and other clinical outcomes in people with baseline ACE and ARB medication use diagnosed with acute lung injury or acute respiratory distress syndrome?

**Search Date**: 18-21 June 2020

**Limitations for Search**

- **Date/Time**: No
- **Language**: No
- **Document Type**: No
- **Publication Status**: No
- **Study Type**: No

**Resources and Number of Results**

| **Resource** | **Time Coverage** | **Search Interface** | **# of Hits^[[1]](#footnote-1)^** |
| --- | --- | --- | --- |
| PubMed | 1946 to 18 June 2020 | PubMed | 194 |
| MEDLINE (ppezv)^[[2]](#footnote-2)^ | 1946 to 18 June 2020 | Ovid | 145 |
| Embase | 1974 to 18 June 2020 | Ovid | 103 |
| ClinicalTrials.gov | to 18 June 2020 | ClinicalTrials.gov | 48 |
| TRIP | to 21 June 2020 | TRIP | 28 |
| Cochrane Library | to 21 June 2020 | Wiley | 27 |
| Total |  |  | 545 |
| Uniques |  |  | 383 |
| Duplicates |  |  | 162 |

**Search Method Section for Paper/Report**

**Materials and Methods**

**Search and Selection of Studies**

On 18 March 2020 PubMed, Ovid Medline, Ovid Embase and ClinicalTrials.gov and on 21 March 2020 TRIP and Cochrane Library, were searched with no date/time, language, document type, publication status or study type limitations.

The search strategies developed by a research information specialist for each resource are as detailed in the previous reports in this series and in **Appendices 1-6**. Only the search in TRIP was limited to the latest year (2020) to reduce retrieval of records already seen in the search results from March; other databases’ search strategies were rerun as given, and any identical records retrieved again for each database search were discarded after import to EndNote, while new duplicates across the different databases were identified and retained in the master EndNote library.

**Appendix 1**

PubMed 1946 to date – new interface, see <https://pubmed.ncbi.nlm.nih.gov/advanced/>

Search completed on 18/06/2020

| **Statement Number** | **Search Statement** | **Results**  **26/03/20** | **Results**  **18/06/20** | **Notes** |
| --- | --- | --- | --- | --- |
| S1 | (((("Orthomyxoviridae"[Mesh] OR "Orthomyxoviridae Infections"[Mesh] OR "Influenza, Human"[Mesh] OR "influenzavirus a"[Mesh] OR "influenzavirus b"[Mesh] OR "influenzavirus c"[Mesh] OR thogotovirus[Mesh])) OR (Orthomyxovir* OR Influenzavir* OR Thogotovir* OR H10N7* OR H10N8* OR H1N1* OR H1N2* OR H2N2* OR H3N2* OR H3N8* OR H5N1* OR H5N2* OR H5N8* OR H7N1* OR H7N2* OR H7N3* OR H7N7* OR H7N9* OR H9N2* OR Grippe)) OR ((HEMAGGLUTININ* AND NEURAMINIDASE*) AND (human OR humans OR virus* OR viral*)) OR ((Orthomyxo* OR Influenza* OR Thogoto* OR Flu) AND (human OR humans OR virus* OR viral*))) | 128175 | 132440 |  |
| S2 | (((Paramyxoviridae[Mesh] OR "Paramyxoviridae Infections"[MESH] OR Pneumovirinae[MESH] OR Pneumovirus[MESH] OR "Pneumovirus Infections"[MESH] OR "Respiratory Syncytial Viruses"[MESH] OR "Respiratory Syncytial Virus, Human"[MESH] OR "Human Respiratory Syncytial Virus"[MESH] OR "Respiratory Syncytial Virus Infections"[MESH] OR "Infections, Respiratory Syncytial Virus"[MESH])) OR (Paramyxovir* OR Pneumovir* OR "Respiratory syncytial virus*" OR RSV* OR "Coryza Agent*")) OR ((Paramyxo* OR Pneumo* OR syncytial*) AND (human OR humans OR virus* OR viral*)) | 116153 | 368568 | 1  Large increase in answers |
| S3 | ((((Respirovirus[Mesh] OR "Parainfluenza virus 1, human"[Mesh] OR "Parainfluenza virus 3, human"[Mesh] OR Rubulavirus[Mesh] OR "Rubulavirus Infections"[Mesh])) OR (Respirovir* OR Rubulavir* OR Parainfluenzavir* OR PIV OR Mumps)) OR ((Respiro* OR Rubula* OR Parainfluenza* OR Para-Influenza* OR "Para Influenza*") AND (human OR humans OR virus* OR viral*))) OR ((Croup* AND (Virus* OR Viral*)) OR (Acute AND Laryngotracheo* AND (Virus* OR Viral* OR Infect*))) | 31172 | 31903 |  |
| S4 | ((Metapneumovirus[MESH]) OR (Metapneumovir* OR "Meta pneumovir*" OR "Meta-pneumovir*")) OR ((Metapneumo* OR "Meta pneumo*" OR "Meta-pneumo*") AND (human OR humans OR virus* OR viral*)) | 2536 | 2236 | ***Fewer*** results than last search! |
| S5 | (((Picornaviridae[MESH] OR Rhinovirus[MESH] OR "Picornaviridae Infections"[Mesh] OR "Common Cold"[Mesh])) OR (Rhinovir* OR Picornavir* OR "Coryza Virus*" OR "Common Cold*")) OR ((Rhino* OR Picorna*) AND (human OR humans OR virus* OR viral*)) | 123492 | 149093 |  |
| S6 | ((((((Coronavirus[Mesh] OR "Coronavirus Infections"[Mesh] OR Betacoronavirus[Mesh] OR "Middle East Respiratory Syndrome Coronavirus"[Mesh] OR "Severe Acute Respiratory Syndrome"[Mesh] OR "SARS Virus"[Mesh] OR "Nsp3 protein, Middle East respiratory syndrome coronavirus" [Supplementary Concept] OR "severe acute respiratory syndrome coronavirus 2"[Supplementary Concept] OR "COVID-19"[Supplementary Concept] OR Nidovirales[MESH] OR Coronaviridae[MESH] OR Alphacoronavirus[MESH] OR "Alphacoronavirus 1"[MESH] OR "Coronavirus 229E, Human"[MESH] OR "Coronavirus NL63, Human"[MESH] OR "Betacoronavirus 1"[MESH] OR "Coronavirus OC43, Human"[MESH] OR "Coronavirus HKU1, Human"[MESH] OR Gammacoronavirus[MESH] OR "Nidovirales Infections"[MESH] OR "Coronaviridae Infections"[MESH])) OR ((Corona* OR Alphacorona* OR "Alpha corona*" OR Alpha-corona* OR Betacorona* OR "Beta corona*" OR Beta-corona* OR Gammacorona* OR "Gamma corona*" OR Gamma-corona* OR Deltacorona* OR "Delta corona*" OR Delta-corona* or Nido*) AND (human OR humans OR virus* OR viral*))) OR (Alphacoronavir* OR "Alpha coronavir*" OR Alpha-coronavir* OR "Beta coronavir*" OR Beta-coronavir* OR Gammacoronavir* OR "Gamma coronavir*" OR Gamma-coronavir* OR Deltacoronavir* OR "Delta coronavir*" OR Delta-coronavir* OR Nidovir*)) OR ("Coronavirus 229E" OR "Coronavirus NL63" or "Transmissible gastroenteritis virus*" OR "TGE virus*" OR "HCoV-HKU1" OR "HCoV-OC43")) OR (Coronavir* OR Betacoronavir* OR SARS OR "Severe Acute Respiratory Syndrome" OR "Severe Acute Respiratory Syndrome 2" OR "SARS-CoV" OR MERS OR "Middle East Respiratory Syndrome" OR "MERS-CoV" OR "Nsp3 protein" OR "COVID-19" OR "SARS-CoV-2" OR "2019 novel coronavirus infection" OR COVID19 OR "coronavirus disease 2019" OR "coronavirus disease-19" OR "2019-nCoV disease" OR "2019 novel coronavirus disease" OR "2019-nCoV infection" OR "Wuhan coronavirus" OR "Wuhan seafood market pneumonia virus" OR COVID2019* OR COVID-2019* OR "coronavirus disease 2019 virus" OR "SARS-CoV-2" OR SARS2 OR "2019-nCoV" OR "2019 novel coronavirus")) | 2419795 | 497359 | 2  ***Many fewer*** results than last search! |
| S7 | (("Pneumonia, Viral"[Mesh] OR Pneumonia[MESH])) OR ("viral pneumonia" OR "atypical PNEUMONIA") | 91619 | 98927 |  |
| S8 | (((Gammaherpesvirinae[Mesh] OR Lymphocryptovirus[Mesh] OR "Herpesvirus 4, Human"[MESH] OR "Epstein-Barr Virus Infections"[MESH])) OR (Lymphocryptovir* OR Gammaherpesvir* OR Gamma-herpesvir* OR "Gamma herpesvir*" OR "Epstein-Barr virus*" OR "Epstein Barr virus*" OR "EBV Infection*" OR "Human Herpesvir*" OR "Herpesvirus 4" OR "Herpes virus 4" OR "Burkitt-Lymphoma Virus*" OR "Burkitt* Lymphoma Virus*" OR "E-B Virus*" OR "E B Virus*" OR "Infectious Mononucleosis Virus*" OR "Burkitt Herpesvirus*" OR "Glandular Fever")) OR ((Lymphocrypto* OR Gammaherpes* OR "Gamma herpes*" OR "Gamma-herpes*"or Herpes*) AND (human OR humans OR virus* OR viral*)) | 145730 | 146915 | 3 |
| S9 | (((Bunyaviridae[MESH] OR Hantavirus[MESH] OR "Hantaan virus"[MESH] OR "Hantavirus Infections"[MESH] OR "Bunyaviridae Infections"[MESH] OR "Hantavirus Pulmonary Syndrome"[MESH] OR "Hemorrhagic Fever with Renal Syndrome"[MESH])) OR (Bunyavir* OR Hantavir* OR "Hantaan virus*" OR "Hanta virus*" OR "Dobrava-Belgrade Virus*" OR "Dobrava Belgrade Virus*" OR "Andes Virus*" OR "Hemorrhagic Fever Virus*" OR "Haemorrhagic Fever Virus*" OR "Nephroso-Nephritis Virus*" OR "Nephroso Nephritis Virus*" OR "HFRS Virus*" OR "Puumala virus*" OR "Seoul virus*" OR "Sin Nombre virus*" OR "Muerto Canyon Virus*" OR "Four Corners Virus*" OR HARDS OR HFRS)) OR ((Bunya* OR Hanta*) AND (human OR humans OR virus* OR viral*)) | 14097 | 14393 |  |
| S10 | (((Alphaherpesvirinae[MESH] OR Varicellovirus[MESH] OR "Herpesvirus 3, Human"[MESH] OR "Varicella Zoster Virus Infection"[MESH] OR "Herpesviridae Infections"[MESH] OR "Varicella Zoster Virus Infection"[MESH] OR Chickenpox[MESH] OR "Herpes Zoster"[MESH] OR "Encephalitis, Varicella Zoster"[Mesh])) OR (Alphaherpesvir* or "Alpha herpesvir*" OR "Alpha-herpesvir*" OR Varicellavir* OR "Varicella-Zoster Virus*" OR "Varicella Zoster Virus*" OR "Herpesvirus 3" OR "Herpes virus 3" OR Chickenpox* OR "Herpes zoster*" OR "HHV 3" OR "HHV-3" OR "VZ Virus*" OR "Herpesvirus Varicellae*" OR "Ocular Herpes zoster Virus*" OR Shingles* OR "Congenital Varicella Syndrome" OR Varicella)) OR ((Varicella* or Alphaherpes* or "Alpha herpes*" OR "Alpha-herpes*") AND (human OR humans OR virus* OR viral*)) | 148244 | 148032 | 4  Minor reduction in number of hits compared to last run of search |
| S11 | (((Betaherpesvirinae[MESH] OR Cytomegalovirus[MESH] OR "Herpesviridae Infections"[MESH] OR "Cytomegalovirus Infections"[MESH] OR "Cytomegalovirus Retinitis"[Mesh])) OR (Cytomegalovir* OR Betaherpesvir* OR "Beta herpesvir*" OR "Beta-herpesvir*" OR "Salivary Gland Virus*" OR "HHV 5" or "HHV-5" OR "Herpesvirus 5" OR "Herpes virus 5" OR "Cytomegalic Inclusion*" OR "Inclusion Disease*")) OR ((Cytomegalo* or Betaherpes* OR "Beta herpes*" OR "Beta-herpes*") AND (human OR humans OR virus* OR viral*)) | 143792 | 142694 | Minor reduction in number of hits compared to last run of search |
| S12 | (((Parvoviridae[MESH] OR Parvovirinae[MESH] OR Bocavirus[MESH] OR "Human bocavirus"[MESH] OR "Parvoviridae Infections"[Mesh])) OR (Bocavir* OR Parvovir*)) OR ((Bocav* OR Parvo*) AND (human OR humans OR virus* OR viral*)) | 20105 | 20426 |  |
| S13 | (((Adenoviridae[MESH] OR Atadenovirus[MESH] OR Mastadenovirus[MESH] OR "Adenoviruses, Human"[MESH] OR "Adenoviridae Infections"[MESH] OR "Adenovirus Infections, Human"[MESH])) OR (Adenovir* OR "Human adenovirus C" OR Mastadenovir* OR Atadenovir* OR "APC Virus*" OR "Pharyngo-Conjunctival Fever" OR "Pharyngo Conjunctival Fever" OR "Pharyngoconjunctival Fever")) OR ((Adeno* OR Atadeno* or Mastadeno*) AND (human OR humans OR virus* OR viral*)) | 290853 | 500856 | Large increase in answers |
| S14 | 1 OR 2 OR 3 OR 4 OR 5 OR 6 OR 7 OR 8 OR 9 OR 10 OR 11 OR 12 OR 13 | 3254856 | 1796359 | Overall, a significant reduction in answers |
| S15 | ((((((("Angiotensin-Converting Enzyme Inhibitors"[Mesh] OR "Angiotensin-Converting Enzyme Inhibitors" [Pharmacological Action] OR "Angiotensin Receptor Antagonists"[Mesh] OR "Angiotensin II Type 1 Receptor Blockers"[Mesh] OR "Angiotensin II Type 1 Receptor Blockers" [Pharmacological Action])) OR (Captopril[Mesh] OR Cilazapril[Mesh] OR Enalapril[Mesh] OR Fosinopril[Mesh] OR Lisinopril[Mesh] OR Perindopril[Mesh] OR Quinapril[Mesh] OR Ramipril[Mesh] OR Teprotid[Mesh] OR Irbesartan[Mesh] OR Losartan[Mesh] OR Telmisartan[Mesh] OR Valsartan[Mesh])) OR (alacepril* OR altiopril* OR benazepril* OR Captopril* OR ceranapril* OR ceronapril* OR Cilazapril* OR deacetylalacepril* OR delapril* OR Enalapril* OR epicaptopril* OR fasidotril* OR foroxymithine OR Fosinopril* OR gemopatril* OR idrapril* OR iletapril* OR imidapril* OR indolapril* OR libenzapril* OR Lisinopril* OR moexipril* OR nitrosocaptopril* OR omapatril* OR pentopril* OR Perindopril* OR pivopril* OR Quinapril* OR Ramipril* OR rentiapril* OR sampatril* OR spirapril* OR temocapril* OR Teprotid* OR trandolapril* OR utibapril* OR zabicipril* OR zofenopril*)) OR (Sartan* OR abitesartan* OR azilsartan* OR candesartan* OR elisartan* OR embusartan* OR enoltasosartan* OR eprosartan* OR fimasartan* OR fonsartan* OR forasartan* OR Irbesartan* OR Losartan* OR milfasartan* OR olmesartan* OR olodanrigan* OR pomisartan* OR pratosartan* OR ripisartan* OR saprisartan* OR sparsentan* OR tasosartan* OR Telmisartan* OR Valsartan* or zolasartan*)) OR ("MDL 100240" OR 57G709* OR "606A compound" OR "A 81988" OR "Abbott 81282" OR "BMS 183920" OR "GR 117289" OR "HN 65021" OR "KD3 671" OR "KR 31080" OR "KRH 594" OR "LR B-081" OR "TH 142177" OR "UR 7247" OR "UR 7280" OR YM358* OR "ZD 7155")) OR (("dipeptidyl carboxypeptidase*" OR "angiotensin converting enzyme*" OR ACE) AND (inhibitor OR inhibitors OR inhibiting OR inhibition OR antagonist OR antagonists OR antagonising OR antagonizing OR antagonism OR blocker OR blockers OR blocking))) OR ((angiotensin* OR AT1 OR AT2) AND (inhibitor OR inhibitors OR inhibiting OR inhibition OR antagonist OR antagonists OR antagonising OR antagonizing OR antagonism OR blocker OR blockers OR blocking)) | 102089 | 106042 | 5 |
| S16 | (("Acute Lung Injury"[MESH] OR "Respiratory Distress Syndrome, Adult"[MESH] OR Pneumonia[MESH] OR "Pneumonia, Viral"[Mesh])) OR ("Respiratory Distress Syndrome*" OR "Acute Lung Injur*" OR "Human ARDS*" OR ARDS OR "Shock Lung*" OR "Lung Shock*" OR pneumonia*) | 262863 | 267644 |  |
| S17 | S14 AND S15 AND S16 | 271 | 423 |  |

|  | **Search Notes online** |
| --- | --- |
| 1 | The asterisk in your search was ignored. You must use 4 or more characters for a wildcard search. Lengthen the root word to search for all endings. Quoted phrases not found: "Human Respiratory Syncytial Virus", "Infections, Respiratory Syncytial Virus" |
| 2 | Quoted phrase not found: "Coronavirus HKU1, Human" |
| 3 | Quoted phrases not found: "Burkitt-Lymphoma Virus*", "Burkitt* Lymphoma Virus*", "Burkitt Herpesvirus*" |
| 4 | Quoted phrase not found: "Ocular Herpes zoster Virus*" |
| 5 | The following terms were not found in PubMed: Teprotid, iletapril*, abitesartan*, elisartan*, pomisartan*, ripisartan* |

Normally in an update, retrieval across the different search statements is expected to increase; however, I have highlighted differences in the retrieval numbers found for some of the search statements above, which appear to be due to the introduction of the new PubMed search interface and search language. There have been many complaints from professional searchers about the impact of the changes on rerun search strategies.

I therefore downloaded the whole final answer set of 423 records; it should be noted that in running the search in March, of the 271 records retrieved, only 7 records were from 2020, now there are 185 from 2020.

From the 423 records retrieved on 18/06/2020, there are 186 new unique records, and 8 new duplicates (of the same article identified in a different database); 8 of the new unique records are from 2012-2019, showing that the changes in the PubMed interface and search language have impacted the retrieval.

The remainder were records that were already identified in the search completed in PubMed on 26/03/2020 (so would not have appeared in this results set if I had felt comfortable in limiting to the date range since the last search).

**Appendix 2**

**Ovid MEDLINE**(R) and Epub Ahead of Print, In-Process & Other Non-Indexed Citations, Daily and Versions(R) <1946 to June 17, 2020> (ppezv)

Search completed on 18/06/2020

| **Statement Number** | **Search Statement** | **Results**  **26/03/20** | **Results**  **18/06/20** |
| --- | --- | --- | --- |
| 1 | exp Orthomyxoviridae/ or exp Orthomyxoviridae Infections/ or exp Influenza, Human/ or exp influenzavirus a/ or exp influenzavirus b/ or exp influenzavirus c/ or exp thogotovirus/ | (80992) | (81814) |
| 2 | (Orthomyxovir* or Influenzavir* or Thogotovir* or H10N7* or H10N8* or H1N1* or H1N2* or H2N2* or H3N2* or H3N8* or H5N1* or H5N2* or H5N8* or H7N1* or H7N2* or H7N3* or H7N7* or H7N9* or H9N2* or Grippe).mp. | (51952) | (52582) |
| 3 | (HEMAGGLUTININ* and NEURAMINIDASE* and (human or humans or virus* or viral*)).mp. | (4149) | (4188) |
| 4 | ((Orthomyxo* or Influenza* or Thogoto* or Flu) and (human or humans or virus* or viral*)).mp. | (126531) | (128262) |
| 5 | exp Paramyxoviridae/ or exp Paramyxoviridae Infections/ or exp Pneumovirinae/ or exp Pneumovirus/ or exp Pneumovirus Infections/ or exp Respiratory Syncytial Viruses/ or exp Respiratory Syncytial Virus, Human/ or exp Human Respiratory Syncytial Virus/ or exp Respiratory Syncytial Virus Infections/ or exp Infections, Respiratory Syncytial Virus/ | (53919) | (54338) |
| 6 | (Paramyxovir* or Pneumovir* or "Respiratory syncytial virus*" or RSV* or "Coryza Agent*").mp. | (25946) | (26356) |
| 7 | ((Paramyxo* or Pneumo* or syncytial*) and (human or humans or virus* or viral*)).mp. | (280765) | (290110) |
| 8 | exp Respirovirus/ or exp Parainfluenza virus 1, human/ or exp Parainfluenza virus 3, human/ or exp Rubulavirus/ or exp Rubulavirus Infections/ | (12782) | (12842) |
| 9 | (Respirovir* or Rubulavir* or Parainfluenzavir* or PIV or Mumps).mp. | (17083) | (17202) |
| 10 | ((Respiro* or Rubula* or Parainfluenza* or Para-Influenza* or "Para Influenza*") and (human or humans or virus* or viral*)).mp. | (12617) | (12710) |
| 11 | (Croup* and (Virus* or Viral*)).mp. | (456) | (458) |
| 12 | (Acute and Laryngotracheo* and (Virus* or Viral* or Infect*)).mp. | (85) | (86) |
| 13 | exp Metapneumovirus/ | (1274) | (1293) |
| 14 | (Metapneumovir* or "Meta pneumovir*" or "Meta-pneumovir*").mp. | (2145) | (2196) |
| 15 | ((Metapneumo* or "Meta pneumo*" or "Meta-pneumo*") and (human or humans or virus* or viral*)).mp. | (2178) | (2226) |
| 16 | exp Picornaviridae/ or exp Rhinovirus/ or exp Picornaviridae Infections/ or exp Common Cold/ | (82212) | (82542) |
| 17 | (Rhinovir* or Picornavir* or "Coryza Virus*" or "Common Cold*").mp. | (15971) | (16231) |
| 18 | ((Rhino* or Picorna*) and (human or humans or virus* or viral*)).mp. | (48148) | (48697) |
| 19 | exp Coronavirus/ or exp Coronavirus Infections/ or exp Betacoronavirus/ or exp Middle East Respiratory Syndrome Coronavirus/ or exp Severe Acute Respiratory Syndrome/ or exp SARS Virus/ or exp Nidovirales/ or exp Coronaviridae/ or exp Alphacoronavirus/ or exp Alphacoronavirus 1/ or exp Coronavirus 229E, Human/ or exp Coronavirus NL63, Human/ or exp Betacoronavirus 1/ or exp Coronavirus OC43, Human/ or exp Coronavirus HKU1, Human/ or exp Gammacoronavirus/ or exp Nidovirales Infections/ or exp Coronaviridae Infections/ | (19856) | (26411) |
| 20 | ((Corona* or Alphacorona* or "Alpha corona*" or Alpha-corona* or Betacorona* or "Beta corona*" or Beta-corona* or Gammacorona* or "Gamma corona*" or Gamma-corona* or Deltacorona* or "Delta corona*" or Delta-corona* or Nido*) and (human or humans or virus* or viral*)).mp. | (447829) | (460418) |
| 21 | (Alphacoronavir* or "Alpha coronavir*" or Alpha-coronavir* or "Beta coronavir*" or Beta-coronavir* or Gammacoronavir* or "Gamma coronavir*" or Gamma-coronavir* or Deltacoronavir* or "Delta coronavir*" or Delta-coronavir* or Nidovir*).mp. | (685) | (797) |
| 22 | ("Coronavirus 229E" or "Coronavirus NL63" or "Transmissible gastroenteritis virus*" or "TGE virus*" or "HCoV-HKU1" or "HCoV-OC43").mp. | (1794) | (1848) |
| 23 | (Coronavir* or Betacoronavir* or SARS or "Severe Acute Respiratory Syndrome" or "Severe Acute Respiratory Syndrome 2" or "SARS-CoV" or MERS or "Middle East Respiratory Syndrome" or "MERS-CoV" or "Nsp3 protein" or "COVID-19" or "SARS-CoV-2" or "2019 novel coronavirus infection" or COVID19 or "coronavirus disease 2019" or "coronavirus disease-19" or "2019-nCoV disease" or "2019 novel coronavirus disease" or "2019-nCoV infection" or "Wuhan coronavirus" or "Wuhan seafood market pneumonia virus" or COVID2019* or COVID-2019* or "coronavirus disease 2019 virus" or "SARS-CoV-2" or SARS2 or "2019-nCoV" or "2019 novel coronavirus").mp. | (24083) | (47930) |
| 24 | exp Pneumonia, Viral/ or exp Pneumonia/ | (90671) | (97681) |
| 25 | ("viral pneumonia" or "atypical PNEUMONIA").mp. | (2302) | (2481) |
| 26 | exp Gammaherpesvirinae/ or exp Lymphocryptovirus/ or exp Herpesvirus 4, Human/ or exp Epstein-Barr Virus Infections/ | (45836) | (46110) |
| 27 | (Lymphocryptovir* or Gammaherpesvir* or Gamma-herpesvir* or "Gamma herpesvir*" or "Epstein-Barr virus*" or "Epstein Barr virus*" or "EBV Infection*" or "Human Herpesvir*" or "Herpesvirus 4" or "Herpes virus 4" or "Burkitt-Lymphoma Virus*" or "Burkitt* Lymphoma Virus*" or "E-B Virus*" or "E B Virus*" or "Infectious Mononucleosis Virus*" or "Burkitt Herpesvirus*" or "Glandular Fever").mp. | (49087) | (49526) |
| 28 | ((Lymphocrypto* or Gammaherpes* or "Gamma herpes*" or "Gamma-herpes*or Herpes*") and (human or humans or virus* or viral*)).mp. | (2369) | (2405) |
| 29 | exp Bunyaviridae/ or exp Hantavirus/ or exp Hantaan virus/ or exp Hantavirus Infections/ or exp Bunyaviridae Infections/ or exp Hantavirus Pulmonary Syndrome/ or exp "Hemorrhagic Fever with Renal Syndrome"/ | (11047) | (11135) |
| 30 | (Bunyavir* or Hantavir* or "Hantaan virus*" or "Hanta virus*" or "Dobrava-Belgrade Virus*" or "Dobrava Belgrade Virus*" or "Andes Virus*" or "Hemorrhagic Fever Virus*" or "Haemorrhagic Fever Virus*" or "Nephroso-Nephritis Virus*" or "Nephroso Nephritis Virus*" or "HFRS Virus*" or "Puumala virus*" or "Seoul virus*" or "Sin Nombre virus*" or "Muerto Canyon Virus*" or "Four Corners Virus*" or HARDS or HFRS).mp. | (9201) | (9290) |
| 31 | ((Bunya* or Hanta*) and (human or humans or virus* or viral*)).mp. | (7620) | (7686) |
| 32 | exp Alphaherpesvirinae/ or exp Varicellavirus/ or exp Herpesvirus 3, Human/ or exp Varicella Zoster Virus Infection/ or exp Herpesviridae Infections/ or exp Varicella Zoster Virus Infection/ or exp Chickenpox/ or exp Herpes Zoster/ or exp Encephalitis, Varicella Zoster/ | (140429) | (141206) |
| 33 | (Alphaherpesvir* or "Alpha herpesvir*" or "Alpha-herpesvir*" or Varicellavir* or "Varicella-Zoster Virus*" or "Varicella Zoster Virus*" or "Herpesvirus 3" or "Herpes virus 3" or Chickenpox* or "Herpes zoster*" or "HHV 3" or "HHV-3" or "VZ Virus*" or "Herpesvirus Varicellae*" or "Ocular Herpes zoster Virus*" or Shingles* or "Congenital Varicella Syndrome" or Varicella).mp. | (29383) | (29682) |
| 34 | ((Varicella* or Alphaherpes* or "Alpha herpes*" or "Alpha-herpes*") and (human or humans or virus* or viral*)).mp. | (15659) | (15839) |
| 35 | exp Betaherpesvirinae/ or exp Cytomegalovirus/ or exp Herpesviridae Infections/ or exp Cytomegalovirus Infections/ or exp Cytomegalovirus Retinitis/ | (128370) | (129102) |
| 36 | (Cytomegalovir* or Betaherpesvir* or "Beta herpesvir*" or "Beta-herpesvir*" or "Salivary Gland Virus*" or "HHV 5" or "HHV-5" or "Herpesvirus 5" or "Herpes virus 5" or "Cytomegalic Inclusion*" or "Inclusion Disease*").mp. | (50496) | (50927) |
| 37 | ((Cytomegalo* or Betaherpes* or "Beta herpes*" or "Beta-herpes*") and (human or humans or virus* or viral*)).mp. | (47871) | (48236) |
| 38 | exp Parvoviridae/ or exp Parvovirinae/ or exp Bocavirus/ or exp Human bocavirus/ or exp Parvoviridae Infections/ | (16519) | (16731) |
| 39 | (Bocavir* or Parvovir*).mp. | (10937) | (11083) |
| 40 | ((Bocav* or Parvo*) and (human or humans or virus* or viral*)).mp. | (11447) | (11585) |
| 41 | exp Adenoviridae/ or exp Atadenovirus/ or exp Mastadenovirus/ or exp Adenoviruses, Human/ or exp Adenoviridae Infections/ or exp Adenovirus Infections, Human/ | (38692) | (38823) |
| 42 | (Adenovir* or "Human adenovirus C" or Mastadenovir* or Atadenovir* or "APC Virus*" or "Pharyngo-Conjunctival Fever" or "Pharyngo Conjunctival Fever" or "Pharyngoconjunctival Fever").mp. | (59752) | (60154) |
| 43 | ((Adeno* or Atadeno* or Mastadeno*) and (human or humans or virus* or viral*)).mp. | (484628) | (488910) |
| 44 | 1 or 2 or 3 or 4 or 5 or 6 or 7 or 8 or 9 or 10 or 11 or 12 or 13 or 14 or 15 or 16 or 17 or 18 or 19 or 20 or 21 or 22 or 23 or 24 or 25 or 26 or 27 or 28 or 29 or 30 or 31 or 32 or 33 or 34 or 35 or 36 or 37 or 38 or 39 or 40 or 41 or 42 or 43 | (1674813) | (1712228) |
| 45 | exp Captopril/ or exp Cilazapril/ or exp Enalapril/ or exp Fosinopril/ or exp Lisinopril/ or exp Perindopril/ or exp Quinapril/ or exp Ramipril/ or exp Teprotid/ | (22362) | (22419) |
| 46 | (alacepril* or altiopril* or benazepril* or Captopril* or ceranapril* or ceronapril* or Cilazapril* or deacetylalacepril* or delapril* or Enalapril* or epicaptopril* or fasidotril* or foroxymithine or Fosinopril* or gemopatril* or idrapril* or iletapril* or imidapril* or indolapril* or libenzapril* or Lisinopril* or moexipril* or nitrosocaptopril* or omapatril* or pentopril* or Perindopril* or pivopril* or Quinapril* or Ramipril* or rentiapril* or sampatril* or spirapril* or temocapril* or Teprotid* or trandolapril* or utibapril* or zabicipril* or zofenopril*).mp. | (31237) | (31378) |
| 47 | exp Angiotensin-Converting Enzyme Inhibitors/ or dipeptidyl carboxypeptidase inhibitor/ or exp Angiotensin Receptor Antagonists/ or exp Angiotensin II Type 1 Receptor Blockers/ | (58275) | (58645) |
| 48 | (("dipeptidyl carboxypeptidase*" or "angiotensin converting enzyme*" or ACE) and (inhibitor or inhibitors or inhibiting or inhibition or antagonist or antagonists or antagonising or antagonizing or antagonism or blocker or blockers or blocking)).mp. | (51789) | (52398) |
| 49 | (ACEI or ACEIs or ARB or ARBs).mp. | (9010) | (9264) |
| 50 | exp Irbesartan/ or exp Losartan/ or exp Telmisartan/ or exp Valsartan/ | (11217) | (11296) |
| 51 | (Sartan* or abitesartan* or azilsartan* or candesartan* or elisartan* or embusartan* or enoltasosartan* or eprosartan* or fimasartan* or fonsartan* or forasartan* or Irbesartan* or Losartan* or milfasartan* or olmesartan* or olodanrigan* or pomisartan* or pratosartan* or ripisartan* or saprisartan* or sparsentan* or tasosartan* or Telmisartan* or Valsartan* or zolasartan*).mp. | (20990) | (21215) |
| 52 | ("MDL 100240" or 57G709* or "606A compound" or "A 81988" or "Abbott 81282" or "BMS 183920" or "GR 117289" or "HN 65021" or "KD3 671" or "KR 31080" or "KRH 594" or "LR B-081" or "TH 142177" or "UR 7247" or "UR 7280" or YM358* or "ZD 7155").mp. | (158) | (158) |
| 53 | ((angiotensin* or AT1 or AT2) and (inhibitor or inhibitors or inhibiting or inhibition or antagonist or antagonists or antagonising or antagonizing or antagonism or blocker or blockers or blocking)).mp. | (82867) | (83797) |
| 54 | 45 or 46 or 47 or 48 or 49 or 50 or 51 or 52 or 53 | (103127) | (104335) |
| 55 | exp Acute Lung Injury/ or exp Respiratory Distress Syndrome, Adult/ or exp Pneumonia/ or exp Pneumonia, Viral/ | (113156) | (120476) |
| 56 | ("Respiratory Distress Syndrome*" or "Acute Lung Injur*" or "Human ARDS*" or ARDS or "Shock Lung*" or "Lung Shock*" or pneumonia*).mp. | (253413) | (264000) |
| 57 | 55 or 56 | (257053) | (267641) |
| 58 | 44 and 54 and 57 | (248) | (423) |

In contrast to the search run on PubMed, the answer sets in Ovid Medline are much more stable, having predictable increases (not surprisingly, search statement 23, which relates to COVID-19, has seen the biggest increase in hits) and no decreases in numbers retrieved.

Note that although 423 records were found in both PubMed and Ovid Medline, they are NOT all equivalent answers.

Importing the 423 final Medline answers to the existing cumulated EndNote library, after the upload of the latest PubMed records, gave 4 new unique records from Medline, plus 141 new duplicates (of the same article identified in a different database, mostly the recent PubMed records); the remainder were records that were already identified from Medline in the search completed on 26/03/2020.

**Appendix 3**

**Embase** 1974 to 2020 June 17

Search completed on 18/06/2020

| **Statement Numbers** | **Search Statements** | **Results**  **26/03/20** | **Results**  **18/06/20** |
| --- | --- | --- | --- |
| 1 | exp Orthomyxoviridae/ or exp Orthomyxoviridae Infection/ or exp Influenza, Human/ or exp influenzavirus a/ or exp influenzavirus b/ or exp influenzavirus c/ or exp thogotovirus/ | (103831) | (105486) |
| 2 | (Orthomyxovir* or Influenzavir* or Thogotovir* or H10N7* or H10N8* or H1N1* or H1N2* or H2N2* or H3N2* or H3N8* or H5N1* or H5N2* or H5N8* or H7N1* or H7N2* or H7N3* or H7N7* or H7N9* or H9N2* or Grippe).ti,ab. | (35612) | (35966) |
| 3 | (HEMAGGLUTININ* adj3 NEURAMINIDASE* adj3 (human or humans or virus* or viral*)).mp. | (525) | (528) |
| 4 | ((Orthomyxo* or Influenza* or Thogoto* or Flu) adj3 (human or humans or virus* or viral*)).mp. | (76695) | (77584) |
| 5 | exp Paramyxoviridae/ or exp Paramyxoviridae Infection/ or exp Pneumovirinae/ or exp Pneumovirus/ or exp Pneumovirus Infection/ or exp Respiratory Syncytial Virus/ or exp Respiratory Syncytial Virus, Human/ or exp Human Respiratory Syncytial Virus/ or exp Respiratory Syncytial Virus Infection/ or exp Infections, Respiratory Syncytial Virus/ | (48899) | (49649) |
| 6 | (Paramyxovir* or Pneumovir* or "Respiratory syncytial virus*" or RSV* or "Coryza Agent*").ti,ab. | (26824) | (27122) |
| 7 | ((Paramyxo* or Pneumo* or syncytial*) adj3 (human or humans or virus* or viral*)).mp. | (31501) | (34038) |
| 8 | exp Respirovirus/ or exp Parainfluenza virus 1, human/ or exp Parainfluenza virus 3, human/ or exp Rubulavirus/ or exp Rubulavirus Infection/ | (11850) | (11991) |
| 9 | (Respirovir* or Rubulavir* or Parainfluenzavir* or PIV or Mumps).ti,ab. | (11300) | (11409) |
| 10 | ((Respiro* or Rubula* or Parainfluenza* or Para-Influenza* or "Para Influenza*") adj3 (human or humans or virus* or viral*)).mp. | (9530) | (9624) |
| 11 | (Croup* adj3 (Virus* or Viral*)).mp. | (149) | (149) |
| 12 | (Acute adj3 Laryngotracheo* adj3 (Virus* or Viral* or Infect*)).mp. | (10) | (10) |
| 13 | exp Metapneumovirus/ | (3409) | (3448) |
| 14 | (Metapneumovir* or "Meta pneumovir*" or "Meta-pneumovir*").ti,ab. | (2943) | (2975) |
| 15 | ((Metapneumo* or "Meta pneumo*" or "Meta-pneumo*") adj3 (human or humans or virus* or viral*)).mp. | (3298) | (3346) |
| 16 | exp Picornaviridae/ or exp Rhinovirus/ or exp Picornaviridae Infection/ or exp Common Cold/ | (77650) | (78427) |
| 17 | (Rhinovir* or Picornavir* or "Coryza Virus*" or "Common Cold*").ti,ab. | (14796) | (14970) |
| 18 | ((Rhino* or Picorna*) adj3 (human or humans or virus* or viral*)).mp. | (7197) | (7258) |
| 19 | exp Coronavirus/ or exp Coronavirus Infection/ or exp Betacoronavirus/ or exp Middle East Respiratory Syndrome Coronavirus/ or exp Severe Acute Respiratory Syndrome/ or exp SARS Virus/ or Nidovirales/ or exp Coronaviridae/ or exp Alphacoronavirus/ or exp Alphacoronavirus 1/ or exp Coronavirus 229E, Human/ or exp Coronavirus NL63, Human/ or exp Betacoronavirus 1/ or exp Coronavirus OC43, Human/ or exp Coronavirus HKU1, Human/ or exp Gammacoronavirus/ or exp Nidovirales Infection/ or exp Coronaviridae Infection/ | (20223) | (24737) |
| 20 | ((Corona* or Alphacorona* or "Alpha corona*" or Alpha-corona* or Betacorona* or "Beta corona*" or Beta-corona* or Gammacorona* or "Gamma corona*" or Gamma-corona* or Deltacorona* or "Delta corona*" or Delta-corona* or Nido*) adj3 (human or humans or virus* or viral*)).mp. | (10237) | (10974) |
| 21 | (Alphacoronavir* or "Alpha coronavir*" or Alpha-coronavir* or "Beta coronavir*" or Beta-coronavir* or Gammacoronavir* or "Gamma coronavir*" or Gamma-coronavir* or Deltacoronavir* or "Delta coronavir*" or Delta-coronavir* or Nidovir*).ti,ab. | (632) | (709) |
| 22 | ("Coronavirus 229E" or "Coronavirus NL63" or "Transmissible gastroenteritis virus*" or "TGE virus*" or "HCoV-HKU1" or "HCoV-OC43").ti,ab. | (1561) | (1597) |
| 23 | (Coronavir* or Betacoronavir* or SARS or "Severe Acute Respiratory Syndrome" or "Severe Acute Respiratory Syndrome 2" or "SARS-CoV" or MERS or "Middle East Respiratory Syndrome" or "MERS-CoV" or "Nsp3 protein" or "COVID-19" or "SARS-CoV-2" or "2019 novel coronavirus infection" or COVID19 or "coronavirus disease 2019" or "coronavirus disease-19" or "2019-nCoV disease" or "2019 novel coronavirus disease" or "2019-nCoV infection" or "Wuhan coronavirus" or "Wuhan seafood market pneumonia virus" or COVID2019* or COVID-2019* or "coronavirus disease 2019 virus" or "SARS-CoV-2" or SARS2 or "2019-nCoV" or "2019 novel coronavirus").ti,ab. | (22539) | (41154) |
| 24 | ("viral pneumonia" or "atypical PNEUMONIA").ti,ab. | (2645) | (2782) |
| 25 | exp Pneumonia, Viral/ or exp Pneumonia/ | (288928) | (295614) |
| 26 | exp Gammaherpesvirinae/ or exp Lymphocryptovirus/ or exp Herpesvirus 4, Human/ or exp Epstein-Barr Virus Infection/ | (64650) | (65396) |
| 27 | (Lymphocryptovir* or Gammaherpesvir* or Gamma-herpesvir* or "Gamma herpesvir*" or "Epstein-Barr virus*" or "Epstein Barr virus*" or "EBV Infection*" or "Human Herpesvir*" or "Herpesvirus 4" or "Herpes virus 4" or "Burkitt-Lymphoma Virus*" or "Burkitt* Lymphoma Virus*" or "E-B Virus*" or "E B Virus*" or "Infectious Mononucleosis Virus*" or "Burkitt Herpesvirus*" or "Glandular Fever").ti,ab. | (46634) | (47070) |
| 28 | ((Lymphocrypto* or Gammaherpes* or "Gamma herpes*" or "Gamma-herpes*or Herpes*") adj3 (human or humans or virus* or viral*)).mp. | (709) | (714) |
| 29 | exp Bunyaviridae/ or exp Hantavirus/ or exp Hantaan virus/ or exp Hantavirus Infection/ or exp Bunyaviridae Infection/ or exp Hantavirus Pulmonary Syndrome/ or exp Hemorrhagic Fever with Renal Syndrome/ | (10961) | (11088) |
| 30 | (Bunyavir* or Hantavir* or "Hantaan virus*" or "Hanta virus*" or "Dobrava-Belgrade Virus*" or "Dobrava Belgrade Virus*" or "Andes Virus*" or "Hemorrhagic Fever Virus*" or "Haemorrhagic Fever Virus*" or "Nephroso-Nephritis Virus*" or "Nephroso Nephritis Virus*" or "HFRS Virus*" or "Puumala virus*" or "Seoul virus*" or "Sin Nombre virus*" or "Muerto Canyon Virus*" or "Four Corners Virus*" or HARDS or HFRS).ti,ab. | (7859) | (7924) |
| 31 | ((Bunya* or Hanta*) adj3 (human or humans or virus* or viral*)).mp. | (2497) | (2511) |
| 32 | exp Alphaherpesvirinae/ or exp Varicellovirus/ or exp Herpesvirus 3, Human/ or exp Varicella Zoster Virus Infection/ or exp Herpesviridae Infection/ or exp Varicella Zoster Virus Infection/ or exp Chickenpox/ or exp Herpes Zoster/ or exp Encephalitis, Varicella Zoster/ | (174546) | (176279) |
| 33 | (Alphaherpesvir* or "Alpha herpesvir*" or "Alpha-herpesvir*" or Varicellavir* or "Varicella-Zoster Virus*" or "Varicella Zoster Virus*" or "Herpesvirus 3" or "Herpes virus 3" or Chickenpox* or "Herpes zoster*" or "HHV 3" or "HHV-3" or "VZ Virus*" or "Herpesvirus Varicellae*" or "Ocular Herpes zoster Virus*" or Shingles* or "Congenital Varicella Syndrome" or Varicella).ti,ab. | (29660) | (29977) |
| 34 | ((Varicella* or Alphaherpes* or "Alpha herpes*" or "Alpha-herpes*") adj3 (human or humans or virus* or viral*)).mp. | (19737) | (20061) |
| 35 | exp Betaherpesvirinae/ or exp Cytomegalovirus/ or exp Herpesviridae Infection/ or exp Cytomegalovirus Infection/ or exp Cytomegalovirus Retinitis/ | (177139) | (178827) |
| 36 | (Cytomegalovir* or Betaherpesvir* or "Beta herpesvir*" or "Beta-herpesvir*" or "Salivary Gland Virus*" or "HHV 5" or "HHV-5" or "Herpesvirus 5" or "Herpes virus 5" or "Cytomegalic Inclusion*" or "Inclusion Disease*").ti,ab. | (52287) | (52720) |
| 37 | ((Cytomegalo* or Betaherpes* or "Beta herpes*" or "Beta-herpes*") adj3 (human or humans or virus* or viral*)).mp. | (17044) | (17188) |
| 38 | exp Parvoviridae/ or exp Parvovirinae/ or exp Bocavirus/ or exp Human bocavirus/ or exp Parvoviridae Infection/ | (17027) | (17360) |
| 39 | (Bocavir* or Parvovir*).ti,ab. | (12115) | (12221) |
| 40 | ((Bocav* or Parvo*) adj3 (human or humans or virus* or viral*)).mp. | (5945) | (6017) |
| 41 | exp Adenoviridae/ or exp Atadenovirus/ or exp Mastadenovirus/ or exp Adenoviruses, Human/ or exp Adenoviridae Infection/ or exp Adenovirus Infections, Human/ | (13006) | (13322) |
| 42 | (Adenovir* or "Human adenovirus C" or Mastadenovir* or Atadenovir* or "APC Virus*" or "Pharyngo-Conjunctival Fever" or "Pharyngo Conjunctival Fever" or "Pharyngoconjunctival Fever").ti,ab. | (63427) | (63885) |
| 43 | ((Adeno* or Atadeno* or Mastadeno*) adj3 (human or humans or virus* or viral*)).mp. | (52467) | (53139) |
| 44 | 129 or 130 or 131 or 132 or 133 or 134 or 135 or 136 or 137 or 138 or 139 or 140 or 141 or 142 or 143 or 144 or 145 or 146 or 147 or 148 or 149 or 150 or 151 or 152 or 153 or 154 or 155 or 156 or 157 or 158 or 159 or 160 or 161 or 162 or 163 or 164 or 165 or 166 or 167 or 168 or 169 or 170 or 171 | (886446) | (914127) |
| 45 | exp Captopril/ or exp Cilazapril/ or exp Enalapril/ or exp Fosinopril/ or exp Lisinopril/ or exp Perindopril/ or exp Quinapril/ or exp Ramipril/ or exp Teprotid/ | (78428) | (78820) |
| 46 | (alacepril* or altiopril* or benazepril* or Captopril* or ceranapril* or ceronapril* or Cilazapril* or deacetylalacepril* or delapril* or Enalapril* or epicaptopril* or fasidotril* or foroxymithine or Fosinopril* or gemopatril* or idrapril* or iletapril* or imidapril* or indolapril* or libenzapril* or Lisinopril* or moexipril* or nitrosocaptopril* or omapatril* or pentopril* or Perindopril* or pivopril* or Quinapril* or Ramipril* or rentiapril* or sampatril* or spirapril* or temocapril* or Teprotid* or trandolapril* or utibapril* or zabicipril* or zofenopril*).ti,ab. | (36672) | (36850) |
| 47 | exp Angiotensin-Converting Enzyme Inhibitor/ or dipeptidyl carboxypeptidase inhibitor/ or exp Angiotensin Receptor Antagonist/ or exp Angiotensin II Type 1 Receptor Blocker/ | (209936) | (211774) |
| 48 | (("dipeptidyl carboxypeptidase*" or "angiotensin converting enzyme*" or ACE) adj3 (inhibitor or inhibitors or inhibiting or inhibition or antagonist or antagonists or antagonising or antagonizing or antagonism or blocker or blockers or blocking)).mp. | (127577) | (128584) |
| 49 | (ACEI or ACEIs or ARB or ARBs).ti,ab. | (17525) | (17865) |
| 50 | exp Irbesartan/ or exp Losartan/ or exp Telmisartan/ or exp Valsartan/ or exp Sartan derivative/ | (39164) | (39523) |
| 51 | (Sartan* or abitesartan* or azilsartan* or candesartan* or elisartan* or embusartan* or enoltasosartan* or eprosartan* or fimasartan* or fonsartan* or forasartan* or Irbesartan* or Losartan* or milfasartan* or olmesartan* or olodanrigan* or pomisartan* or pratosartan* or ripisartan* or saprisartan* or sparsentan* or tasosartan* or Telmisartan* or Valsartan* or zolasartan*).ti,ab. | (28406) | (28713) |
| 52 | ("MDL 100240" or 57G709* or "606A compound" or "A 81988" or "Abbott 81282" or "BMS 183920" or "GR 117289" or "HN 65021" or "KD3 671" or "KR 31080" or "KRH 594" or "LR B-081" or "TH 142177" or "UR 7247" or "UR 7280" or YM358* or "ZD 7155").ti,ab. | (97) | (97) |
| 53 | ((angiotensin* or AT1 or AT2) adj3 (inhibitor or inhibitors or inhibiting or inhibition or antagonist or antagonists or antagonising or antagonizing or antagonism or blocker or blockers or blocking)).mp. | (89486) | (90501) |
| 54 | 45 or 46 or 47 or 48 or 49 or 50 or 51 or 52 or 53 | (226723) | (228648) |
| 55 | exp *Acute Lung Injury/ or exp *Respiratory Distress Syndrome, Adult/ or exp *Pneumonia/ or exp *Pneumonia, Viral/ | (122252) | (124449) |
| 56 | ("Respiratory Distress Syndrome*" or "Acute Lung Injur*" or "Human ARDS*" or ARDS or "Shock Lung*" or "Lung Shock*" or pneumonia*).mp. | (450103) | (459454) |
| 57 | 55 or 56 | (478117) | (471687) |
| 58 | 44 and 54 and 57 | (2404) | (2562) |

Similarly to the search run on Ovid Medline, the answer sets in Ovid Embase are also stable (again, search statement 23, which relates to COVID-19, has seen the biggest increase in hits) and no decreases in numbers retrieved.

Importing the 2562 final Embase answers to the existing cumulated EndNote library gave 100 new unique records from Embase (of which 20 are identified as “case reports”), plus 3 new duplicates (of the same article identified in a different database); the remainder were records that were already identified from Embase in the search completed on 26/03/2020. Of the 100 new unique records, 28 are pre-2020, so I suspect that EMTREE indexing is being updated for older records on coronaviruses.

**Appendix 4**

ClinicalTrials.gov; search completed on 18/06/2020

| **Condition or disease** | **AND Other terms** | **AND Intervention/treatment** | **Studies found 26/03/20^[[3]](#footnote-3)^** | **Studies found 18/06/20^1^** |
| --- | --- | --- | --- | --- |
| NOT cancer | Acute Lung Injury OR Adult Respiratory Distress Syndrome OR Acute Respiratory Distress Syndrome OR Pneumonia | (Sartan OR abitesartan OR azilsartan OR candesartan OR elisartan OR embusartan OR enoltasosartan OR eprosartan OR fimasartan OR fonsartan OR forasartan OR Irbesartan OR Losartan OR milfasartan OR olmesartan OR olodanrigan) OR (pomisartan OR pratosartan OR ripisartan OR saprisartan OR sparsentan OR tasosartan OR Telmisartan OR Valsartan or zolasartan) | 5 | 5 |
| NOT cancer | Acute Lung Injury OR Adult Respiratory Distress Syndrome OR Acute Respiratory Distress Syndrome OR Pneumonia | (alacepril OR altiopril OR benazepril OR Captopril OR ceranapril OR ceronapril OR Cilazapril OR deacetylalacepril OR delapril OR Enalapril OR epicaptopril OR fasidotril OR foroxymithine OR Fosinopril OR gemopatril OR idrapril OR iletapril OR imidapril) OR (indolapril OR libenzapril OR Lisinopril OR moexipril OR nitrosocaptopril OR omapatril OR pentopril OR Perindopril OR pivopril OR Quinapril OR Ramipril OR rentiapril OR sampatril OR spirapril OR temocapril OR Teprotid OR trandolapril OR utibapril) OR (zabicipril OR zofenopril OR dipeptidyl carboxypeptidase inhibitor) | 3 | 8 |
| Coronavirus Infection OR Orthomyxoviridae infections OR Paramyxoviridae infections OR Respiratory Syncytial Virus infections OR Respiratory Tract Infections OR Virus Diseases OR Infections OR RNA Virus Infections | Acute Lung Injury OR Adult Respiratory Distress Syndrome OR Acute Respiratory Distress Syndrome OR Pneumonia | Angiotensin-Converting Enzyme OR Angiotensin-Converting Enzyme Inhibitors OR Angiotensin Receptor Antagonists OR Angiotensin receptor blockers OR Angiotensin I Converting Enzyme OR Angiotensin I Converting Enzyme, Human | 4 | 4 |
| H10N7 OR H10N8 OR H1N1 OR H1N2 OR H2N2 OR H3N2 OR H3N8 OR H5N1 OR H5N2 OR H5N8 OR H7N1 OR H7N2 OR H7N3 OR H7N7 OR H7N9 OR H9N2 |  | Angiotensin-Converting Enzyme OR Angiotensin-Converting Enzyme Inhibitors OR Angiotensin Receptor Antagonists OR Angiotensin receptor blockers OR Angiotensin I Converting Enzyme OR Angiotensin I Converting Enzyme, Human | 0 | 0 |
| Influenza OR Influenza A OR Influenza, Human OR Influenza Type B OR Influenza -Like Illness OR Influenza A Virus Infection OR Influenza with Pneumonia OR Influenza Viral Infections OR Influenza A H3N2 |  | Angiotensin-Converting Enzyme OR Angiotensin-Converting Enzyme Inhibitors OR Angiotensin Receptor Antagonists OR Angiotensin receptor blockers OR Angiotensin I Converting Enzyme OR Angiotensin I Converting Enzyme, Human | 2 | 2 |
| Parainfluenza OR Parainfluenza 1 Infection OR Mumps OR Croup OR Metapneumovirus OR Picornaviridae Infections OR Rhinovirus OR Coryza |  | Angiotensin-Converting Enzyme OR Angiotensin-Converting Enzyme Inhibitors OR Angiotensin Receptor Antagonists OR Angiotensin receptor blockers OR Angiotensin I Converting Enzyme OR Angiotensin I Converting Enzyme, Human | 5 | 5 |
| SARS OR Severe Acute Respiratory Syndrome OR Severe Acute Respiratory Syndrome 2 OR SARS-CoV OR MERS OR Middle East Respiratory Syndrome OR MERS-CoV OR Nsp3 protein OR COVID-19 OR SARS-CoV-2 |  | Angiotensin-Converting Enzyme OR Angiotensin-Converting Enzyme Inhibitors OR Angiotensin Receptor Antagonists OR Angiotensin receptor blockers OR Angiotensin I Converting Enzyme OR Angiotensin I Converting Enzyme, Human | 8 | 49 |
| 2019 novel coronavirus infection OR COVID19 OR coronavirus disease 2019 OR coronavirus disease-19 OR 2019-nCoV disease OR 2019 novel coronavirus disease OR 2019-nCoV infection OR Wuhan coronavirus |  | Angiotensin-Converting Enzyme OR Angiotensin-Converting Enzyme Inhibitors OR Angiotensin Receptor Antagonists OR Angiotensin receptor blockers OR Angiotensin I Converting Enzyme OR Angiotensin I Converting Enzyme, Human | 7 | 48 |
| Wuhan seafood market pneumonia virus OR COVID2019* OR COVID-2019* OR coronavirus disease 2019 virus OR SARS-CoV-2 OR SARS2 OR 2019-nCoV OR 2019 novel coronavirus |  | Angiotensin-Converting Enzyme OR Angiotensin-Converting Enzyme Inhibitors OR Angiotensin Receptor Antagonists OR Angiotensin receptor blockers OR Angiotensin I Converting Enzyme OR Angiotensin I Converting Enzyme, Human | 7 | 48 |
| Gammaherpesviral Mononucleosis OR Herpesvirus Infection OR Herpesvirus Keratitis OR Herpesvirus Hominis Disease OR Herpesvirus 4 Infections, Human OR Herpesviridae Infections OR Herpesviral Encephalitis OR Herpes Zoster |  | Angiotensin-Converting Enzyme OR Angiotensin-Converting Enzyme Inhibitors OR Angiotensin Receptor Antagonists OR Angiotensin receptor blockers OR Angiotensin I Converting Enzyme OR Angiotensin I Converting Enzyme, Human | 1 | 1 |
| Epstein-Barr Virus Infections OR Burkitt-Lymphoma OR Hanta Virus OR Hantavirus Infection OR Hanta Virus Pulmonary Syndrome OR Hantavirus Pulmonary Syndrome OR Hanta Virus Infection (Korean) |  | Angiotensin-Converting Enzyme OR Angiotensin-Converting Enzyme Inhibitors OR Angiotensin Receptor Antagonists OR Angiotensin receptor blockers OR Angiotensin I Converting Enzyme OR Angiotensin I Converting Enzyme, Human | 1 | 1 |
| Varicella OR Varicella Zoster OR Varicella Zoster Virus Infection OR Varicella Keratitis OR Chickenpox |  | Angiotensin-Converting Enzyme OR Angiotensin-Converting Enzyme Inhibitors OR Angiotensin Receptor Antagonists OR Angiotensin receptor blockers OR Angiotensin I Converting Enzyme OR Angiotensin I Converting Enzyme, Human | 0 | 0 |
| Cytomegalovirus Infections OR Cytomegalovirus Retinitis OR Cytomegalovirus Viremia OR Cytomegalovirus Congenital OR Cytomegalovirus Colitis OR Cytomegalovirus Infection Reactivation OR Parvovirus B19 Infection |  | Angiotensin-Converting Enzyme OR Angiotensin-Converting Enzyme Inhibitors OR Angiotensin Receptor Antagonists OR Angiotensin receptor blockers OR Angiotensin I Converting Enzyme OR Angiotensin I Converting Enzyme, Human | 1 | 1 |
| Adenoviridae Infection OR Adenovirus OR Adenoviral Conjunctivitis OR Adenovirus Disease OR Adenovirus Infection OR Adenoviral Infection OR Adenoviral Keratoconjunctivitis OR Adenovirus Infections, Human |  | Angiotensin-Converting Enzyme OR Angiotensin-Converting Enzyme Inhibitors OR Angiotensin Receptor Antagonists OR Angiotensin receptor blockers OR Angiotensin I Converting Enzyme OR Angiotensin I Converting Enzyme, Human | 0 | 0 |
| NOT cancer | Acute Lung Injury OR Adult Respiratory Distress Syndrome OR Acute Respiratory Distress Syndrome OR Pneumonia | Angiotensin I Converting Enzyme Inhibitors OR Angiotensin I Converting Enzyme Wt Allele OR Angiotensin II Blockers OR Angiotensin II Blockers Azilsartan Medoxomil OR Angiotensin II Blockers Telmisartan + Amlodipine | 6 | 6 |
| NOT cancer | Acute Lung Injury OR Adult Respiratory Distress Syndrome OR Acute Respiratory Distress Syndrome OR Pneumonia | Angiotensin II Receptor 2-Interacting Protein OR Angiotensin II Receptor Antagonists OR Angiotensin II Receptor Blocker OR Angiotensin II type 1 receptor antagonists OR Angiotensin II Type 1 Receptor Blockers | 6 | 6 |
| NOT cancer | Acute Lung Injury OR Adult Respiratory Distress Syndrome OR Acute Respiratory Distress Syndrome OR Pneumonia | ACE inhibitor OR Ace Inhibitors Moexipril OR Ace Inhibitors Fosinopril OR Ace Inhibitors Quinapril Hcl OR Ace Inhibitors Perindopril OR Ace Inhibitors Perindopril Arginine OR ACE Inhibitors + Thiazides: Lisinopril + Hydrochlorothiazide | 6 | 6 |

There is a significant increase of Clinical Trials records found relating to COVID-19 (see highlighted sets above).

A total of 48 new trial records were identified, of which 46 are new unique record, and 2 are duplicates (previously identified in ClinicalTrials.gov or Cochrane Library, and with updated company “author” details).

**Appendix 5**

TRIP; search completed on 26 March 2020

| **Search statements^[[4]](#footnote-4)^** | **Results from 26/03/20** | **Search statements^[[5]](#footnote-5)^** | **Results from 21/06/20** |
| --- | --- | --- | --- |
| Title(ACE inhibitor OR ACE Inhibitors) | 1045 | ((title:ACE inhibitor) OR (title:ACE Inhibitors)) from:2020 | 5^[[6]](#footnote-6)^ |
| Population(Coronavirus Infection OR Orthomyxoviridae infections OR Paramyxoviridae infections OR Respiratory Syncytial Virus infections OR Respiratory Tract Infections OR Virus Diseases OR Infections OR RNA Virus Infections) AND Intervention(Angiotensin-Converting Enzyme OR Angiotensin-Converting Enzyme Inhibitors OR Angiotensin Receptor Antagonists OR Angiotensin receptor blockers OR Angiotensin I Converting Enzyme OR Angiotensin I Converting Enzyme, Human) | 3058 | Population(Coronavirus Infection OR Orthomyxoviridae infections OR Paramyxoviridae infections OR Respiratory Syncytial Virus infections OR Respiratory Tract Infections OR Virus Diseases OR Infections OR RNA Virus Infections) AND Intervention(Angiotensin-Converting Enzyme OR Angiotensin-Converting Enzyme Inhibitors OR Angiotensin Receptor Antagonists OR Angiotensin receptor blockers OR Angiotensin I Converting Enzyme OR Angiotensin I Converting Enzyme, Human) from:2020 | 37^[[7]](#footnote-7)^ |
| Population(SARS OR Severe Acute Respiratory Syndrome OR Severe Acute Respiratory Syndrome 2 OR SARS-CoV OR MERS OR Middle East Respiratory Syndrome OR MERS-CoV OR Nsp3 protein OR COVID-19 OR SARS-CoV-2 OR 2019 novel coronavirus infection OR COVID19 OR coronavirus disease 2019 OR coronavirus disease-19 OR 2019-nCoV disease OR 2019 novel coronavirus disease OR 2019-nCoV infection OR Wuhan coronavirus OR Wuhan seafood market pneumonia virus OR COVID2019 OR COVID-2019 OR coronavirus disease 2019 virus OR SARS-CoV-2 OR SARS2 OR 2019-nCoV OR 2019 novel coronavirus OR Coronavirus Infection OR Orthomyxoviridae infections OR Paramyxoviridae infections OR Respiratory Syncytial Virus infections OR Respiratory Tract Infections OR Virus Diseases OR Infections OR RNA Virus Infections) AND Intervention(Angiotensin Receptor Antagonists OR Angiotensin receptor blockers OR Angiotensin I Converting Enzyme OR Angiotensin II Receptor 2-Interacting Protein OR Angiotensin II Receptor Antagonists OR Angiotensin II Receptor Blocker OR Angiotensin II type 1 receptor antagonists OR Angiotensin II Type 1 Receptor Blockers) AND Outcome(Acute Lung Injury OR Adult Respiratory Distress Syndrome OR Acute Respiratory Distress Syndrome OR Pneumonia) | 431 | Population(SARS OR Severe Acute Respiratory Syndrome OR Severe Acute Respiratory Syndrome 2 OR SARS-CoV OR MERS OR Middle East Respiratory Syndrome OR MERS-CoV OR Nsp3 protein OR COVID-19 OR SARS-CoV-2 OR 2019 novel coronavirus infection OR COVID19 OR coronavirus disease 2019 OR coronavirus disease-19 OR 2019-nCoV disease OR 2019 novel coronavirus disease OR 2019-nCoV infection OR Wuhan coronavirus OR Wuhan seafood market pneumonia virus OR COVID2019 OR COVID-2019 OR coronavirus disease 2019 virus OR SARS-CoV-2 OR SARS2 OR 2019-nCoV OR 2019 novel coronavirus OR Coronavirus Infection OR Orthomyxoviridae infections OR Paramyxoviridae infections OR Respiratory Syncytial Virus infections OR Respiratory Tract Infections OR Virus Diseases OR Infections OR RNA Virus Infections) AND Intervention(Angiotensin Receptor Antagonists OR Angiotensin receptor blockers OR Angiotensin I Converting Enzyme OR Angiotensin II Receptor 2-Interacting Protein OR Angiotensin II Receptor Antagonists OR Angiotensin II Receptor Blocker OR Angiotensin II type 1 receptor antagonists OR Angiotensin II Type 1 Receptor Blockers) from:2020 | 37^[[8]](#footnote-8)^ |
| Population (SARS OR Severe Acute Respiratory Syndrome OR Severe Acute Respiratory Syndrome 2 OR SARS-CoV OR MERS OR Middle East Respiratory Syndrome OR MERS-CoV OR Nsp3 protein OR COVID-19 OR SARS-CoV-2 OR 2019 novel coronavirus infection OR COVID19 OR coronavirus disease 2019 OR coronavirus disease-19 OR 2019-nCoV disease OR 2019 novel coronavirus disease OR 2019-nCoV infection OR Wuhan coronavirus OR Wuhan seafood market pneumonia virus OR COVID2019 OR COVID-2019 OR coronavirus disease 2019 virus OR SARS-CoV-2 OR SARS2 OR 2019-nCoV OR 2019 novel coronavirus OR Coronavirus Infection OR Orthomyxoviridae infections OR Paramyxoviridae infections OR Respiratory Syncytial Virus infections OR Respiratory Tract Infections OR Virus Diseases OR Infections OR RNA Virus Infections) AND Intervention(Sartan OR abitesartan OR azilsartan OR candesartan OR elisartan OR embusartan OR enoltasosartan OR eprosartan OR fimasartan OR fonsartan OR forasartan OR Irbesartan OR Losartan OR milfasartan OR olmesartan OR olodanrigan) AND Outcome(Acute Lung Injury OR Adult Respiratory Distress Syndrome OR Acute Respiratory Distress Syndrome OR Pneumonia) | 28 | Population (SARS OR Severe Acute Respiratory Syndrome OR Severe Acute Respiratory Syndrome 2 OR SARS-CoV OR MERS OR Middle East Respiratory Syndrome OR MERS-CoV OR Nsp3 protein OR COVID-19 OR SARS-CoV-2 OR 2019 novel coronavirus infection OR COVID19 OR coronavirus disease 2019 OR coronavirus disease-19 OR 2019-nCoV disease OR 2019 novel coronavirus disease OR 2019-nCoV infection OR Wuhan coronavirus OR Wuhan seafood market pneumonia virus OR COVID2019 OR COVID-2019 OR coronavirus disease 2019 virus OR SARS-CoV-2 OR SARS2 OR 2019-nCoV OR 2019 novel coronavirus OR Coronavirus Infection OR Orthomyxoviridae infections OR Paramyxoviridae infections OR Respiratory Syncytial Virus infections OR Respiratory Tract Infections OR Virus Diseases OR Infections OR RNA Virus Infections) AND Intervention(Sartan OR abitesartan OR azilsartan OR candesartan OR elisartan OR embusartan OR enoltasosartan OR eprosartan OR fimasartan OR fonsartan OR forasartan OR Irbesartan OR Losartan OR milfasartan OR olmesartan OR olodanrigan) AND Outcome(Acute Lung Injury OR Adult Respiratory Distress Syndrome OR Acute Respiratory Distress Syndrome OR Pneumonia) from 2020 | 18^[[9]](#footnote-9)^ |

**Appendix 6**

Cochrane Library; completed on 21/06/2020

| **Search statement^[[10]](#footnote-10)^** | **Results found 29/03/20** | **Results found 21/06/20^[[11]](#footnote-11)^** |
| --- | --- | --- |
| Cochrane Reviews matching respiratory distress AND angiotensin receptor antagonist in All Text | 6 | 6 |
| Trials matching respiratory distress AND angiotensin receptor antagonist in All Text | 4 | 8 |
| Cochrane Reviews matching acute lung injury AND angiotensin receptor antagonist in All Text | 9 | 9 |
| Trials matching acute lung injury AND angiotensin receptor antagonist in All Text | 4 | 18 |
| Cochrane Reviews matching pneumonia AND angiotensin receptor antagonist in All Text | 14 | 14 |
| Trials matching pneumonia AND angiotensin receptor antagonist in All Text | 12 | 12 |
| Cochrane Reviews matching MeSH descriptor: [Angiotensin Receptor Antagonists] | 15 | 16 |
| Trials matching MeSH descriptor: [Angiotensin Receptor Antagonists] explode all trees | 2095 | 2106 |
| Cochrane Reviews matching MeSH descriptor: [Angiotensin-Converting Enzyme Inhibitors] explode all trees | 27 | 28 |
| Trials matching MeSH descriptor: [Angiotensin-Converting Enzyme Inhibitors] explode all trees | 3938 | 3952 |
| Cochrane Reviews matching respiratory distress AND Angiotensin-Converting Enzyme Inhibitors in All Text | 11 | 11^[[12]](#footnote-12)^ |
| Trials matching respiratory distress AND Angiotensin-Converting Enzyme Inhibitors in All Text - (Word variations have been searched) | 10 | 14 |
| Cochrane Reviews matching acute lung injury AND Angiotensin-Converting Enzyme Inhibitors in All Text | 18 | 19 |
| Trials matching acute lung injury AND Angiotensin-Converting Enzyme Inhibitors in All Text | 5 | 21 |
| Cochrane Reviews matching pneumonia AND Angiotensin-Converting Enzyme Inhibitors in All Text | 27 | 28^[[13]](#footnote-13)^ |
| Trials matching pneumonia AND Angiotensin-Converting Enzyme Inhibitors in All Text | 20 | 20 |

**Appendix 7** Chinese literature search

新型冠状病毒+新型冠状病毒感染+中东呼吸道综合征+严重呼吸窘迫综合征冠状病毒+SARS病毒+严重呼吸窘迫综合征2+COVID-19+巣状病毒网+冠状病毒科+人冠状病毒+严重急性呼吸道综合征2+SARS-CoV+SARS-CoV-2+2019 新型冠状病毒感染+冠状病毒疾病 2019+冠状病毒-19+2019-nCoV 疾病+2019新型冠状病毒疾病+2019新型冠状病毒肺炎+2019-nCoV感染+武汉冠状病毒

血管紧张素转化酶抑制剂+血管紧张素受体拮抗剂+血管紧张素受体抑制剂+血管紧张素II型受体拮抗剂+卡托普利+西那普利+依那普利+福辛普利+赖诺普利+培哚普利+雷米普利+厄贝沙坦+氯沙坦+替米沙坦+缬沙坦+坎地沙坦+奥美沙坦+坎地沙坦+阿拉普利+阿速普利+本那普利+施瑞普利+西罗普利+地那普利+依那普利+法西多曲+甲羟米辛+伊曲普利+咪达普利+赖苯普利+莫西普利+喷托普利+匹伏普利+喹那普利+伦唑普利+螺普利+替莫普利+群多普利+乌替普利+扎普利+佐芬普利+沙坦+阿比沙坦+阿齐沙坦+依利沙坦+恩布沙坦+依普罗沙坦+非马沙坦+福拉沙坦+普拉沙坦+利匹沙坦+沙普利沙坦+他索沙坦

|  | CNKI | 万方 | CBM |
| --- | --- | --- | --- |
| 检索数 | 43 | 21 | 55 |
| 去重后 | 43 | 4 | 26 |
| 合计 | 73 | | |

2021-5-8

1. CNKI（期刊、学位、会议，中英文扩展：否）

(SU%=新型冠状病毒+"SARS-CoV"+"SARS-CoV-2"+"2019-nCoV"+"COVID-19"+中东呼吸道综合征+MERS+严重呼吸窘迫综合征冠状病毒+SARS +"严重呼吸窘迫综合征2"+巣状病毒网+冠状病毒科+冠状病毒+人冠状病毒+"严重急性呼吸道综合征2" OR TKA=新型冠状病毒+"SARS-CoV"+"SARS-CoV-2"+"2019-nCoV"+"COVID-19"+中东呼吸道综合征+MERS+严重呼吸窘迫综合征冠状病毒+SARS +"严重呼吸窘迫综合征2"+巣状病毒网+冠状病毒科+冠状病毒+人冠状病毒+"严重急性呼吸道综合征2") AND (SU%=血管紧张素转化酶抑制剂+血管紧张素转化酶拮抗剂+血管紧张素受体拮抗剂+血管紧张素受体抑制剂+血管紧张素拮抗剂+血管紧张素抑制剂+卡托普利+依那普利+福辛普利+福辛普利钠+赖诺普利+培哚普利+雷米普利+厄贝沙坦+氯沙坦+替米沙坦+缬沙坦+坎地沙坦+奥美沙坦+咪达普利+沙坦+沙坦类 OR TKA=血管紧张素转化酶抑制剂+血管紧张素转化酶拮抗剂+血管紧张素受体拮抗剂+血管紧张素受体抑制剂+血管紧张素拮抗剂+血管紧张素抑制剂+卡托普利+依那普利+福辛普利+福辛普利钠+赖诺普利+培哚普利+雷米普利+厄贝沙坦+氯沙坦+替米沙坦+缬沙坦+坎地沙坦+奥美沙坦+咪达普利+沙坦+沙坦类) 43

1. 万方（期刊、学位、会议）

主题:("新型冠状病毒" OR "SARS-CoV" OR "SARS-CoV-2" OR "2019-nCoV" OR "COVID-19" OR "中东呼吸道综合征" OR MERS OR "严重呼吸窘迫综合征冠状病毒" OR SARS OR "严重呼吸窘迫综合征2" OR "巣状病毒" OR "冠状病毒" OR "严重急性呼吸道综合征2") and 主题:("血管紧张素转化酶抑制剂" OR "血管紧张素转化酶拮抗剂" OR "血管紧张素受体拮抗剂" OR "血管紧张素受体抑制剂" OR "血管紧张素拮抗剂" OR "血管紧张素抑制剂" OR "卡托普利" OR "依那普利" OR "福辛普利" OR "赖诺普利" OR "培哚普利" OR "雷米普利" OR "厄贝沙坦" OR "氯沙坦" OR "替米沙坦" OR "缬沙坦" OR "坎地沙坦" OR "奥美沙坦" OR "咪达普利" OR "沙坦") 21

1. CBM

( "新型冠状病毒"[常用字段:智能] OR "SARS-CoV"[常用字段:智能] OR "SARS-CoV-2"[常用字段:智能] OR "2019-nCoV"[常用字段:智能] OR "COVID-19"[常用字段:智能] OR "中东呼吸道综合征"[常用字段:智能] OR "MERS"[常用字段:智能] OR "严重呼吸窘迫综合征冠状病毒"[常用字段:智能] OR "SARS "[常用字段:智能] OR "严重呼吸窘迫综合征2"[常用字段:智能] OR "巣状病毒网"[常用字段:智能] OR "冠状病毒科"[常用字段:智能] OR "冠状病毒"[常用字段:智能] OR "人冠状病毒"[常用字段:智能] OR "严重急性呼吸道综合征2"[常用字段:智能]) AND ( "血管紧张素转化酶抑制剂"[常用字段:智能] OR "血管紧张素转化酶拮抗剂"[常用字段:智能] OR "血管紧张素受体拮抗剂"[常用字段:智能] OR "血管紧张素受体抑制剂"[常用字段:智能] OR "血管紧张素拮抗剂"[常用字段:智能] OR "血管紧张素抑制剂"[常用字段:智能] OR "卡托普利"[常用字段:智能] OR "依那普利"[常用字段:智能] OR "福辛普利"[常用字段:智能] OR "福辛普利钠"[常用字段:智能] OR "赖诺普利"[常用字段:智能] OR "培哚普利"[常用字段:智能] OR "雷米普利"[常用字段:智能] OR "厄贝沙坦"[常用字段:智能] OR "氯沙坦"[常用字段:智能] OR "替米沙坦"[常用字段:智能] OR "缬沙坦"[常用字段:智能] OR "坎地沙坦"[常用字段:智能] OR "奥美沙坦"[常用字段:智能] OR "咪达普利"[常用字段:智能] OR "沙坦"[常用字段:智能] OR "沙坦类"

1. See Appendices 1-6 for details. [↑](#footnote-ref-1)
2. ppezv = Ovid MEDLINE(R) and Epub Ahead of Print, In-Process & Other Non-Indexed Citations, Daily and Versions(R). [↑](#footnote-ref-2)
3. The search statements may have duplicate answers in their results sets. [↑](#footnote-ref-3)
4. The search statements may have duplicate answers in their results sets; the sets were reviewed individually for possibly relevant records by selecting subsets of:

   Key primary research

   Systematic reviews

   Controlled trials

   Primary research

   Ongoing systematic reviews [↑](#footnote-ref-4)
5. The search statements were limited to 2020 and reviewed as before [↑](#footnote-ref-5)
6. Of this set, 2 new SRs identified, 1 previously found; other records not relevant [↑](#footnote-ref-6)
7. Of this set, 4 new SRs identified; 8 evidence based synopses, 1 previously found, 7 not relevant; other records not relevant [↑](#footnote-ref-7)
8. This strategy would not run with the “Outcome” included. Of this set, 1 new SR identified, other records not relevant [↑](#footnote-ref-8)
9. Of this set, 2 new SRs identified; other records not relevant [↑](#footnote-ref-9)
10. For All Text queries, Word variations have been searched; for MeSH descriptor searches, explode all trees is applied [↑](#footnote-ref-10)
11. All results from these individual statements were imported to EndNote and identical records from the different search statements removed, to give a total of 27 additional records; 7 are duplicates, 20 are new unique records [↑](#footnote-ref-11)
12. A Cochrane Clinical Answer reference was retrieved in addition to the Cochrane Reviews [↑](#footnote-ref-12)
13. A Protocol (which does not appear to be relevant) and another Cochrane Clinical Answer were retrieved [↑](#footnote-ref-13)
