# Supplementary material for: Influence of angiotensin converting enzyme inhibitors/angiotensin receptor blockers on the risk of all‐cause mortality and other clinical outcomes in patients with confirmed COVID‐19: A systemic review and meta‐analysis
Source: J Clin Hypertens (Greenwich). 2021 Jul 28;23(9):1651–63. doi: 10.1111/jch.14329 (PMC8420264; doi:10.1111/jch.14329)
Supplement: Supplementary file 2 — Supporting material [file JCH-23-1651-s001.docx]

| **ROBINS-tool** | **Overall Judgement** | Bias due to confounding | Bias in selection of participants into study | Bias in classification of interventions | Bias due to deviation from intended intervention | Bias due to missing data | Bias in measurement of outcomes | Bias in selection of the reported result |
| --- | --- | --- | --- | --- | --- | --- | --- | --- |
| Meng 2020 | Moderate risk | Moderate risk | Low risk | Low risk | Low risk | Low risk | Low risk | Low risk |
| Huang 2020 | Serious risk | Serious risk | Moderate risk | Low risk | Low risk | Low risk | Low risk | Low risk |
| Richardson 2020 | Low risk | Low risk | Low risk | Low risk | Low risk | Low risk | Low risk | Low risk |
| Mehta 2020 | Moderate risk | Moderate risk | Low risk | Moderate risk | Low risk | Low risk | Low risk | Low risk |
| Jung 2020 | Low risk | Low risk | Low risk | Low risk | Low risk | Low risk | Low risk | Low risk |
| Bean 2020 | Low risk | Low risk | Low risk | Low risk | Low risk | Low risk | Low risk | Low risk |
| Zhang 2020 | Moderate risk | Moderate risk | Low risk | Low risk | Low risk | Low risk | Low risk | Low risk |
| Otero 2020 | Low risk | Low risk | Low risk | Low risk | Low risk | Low risk | Low risk | Low risk |
| Gao 2020 | Low risk | Low risk | Low risk | Low risk | Low risk | Low risk | Low risk | Low risk |
| Imam 2020 | Moderate risk | Low risk | Low risk | Moderate risk | Low risk | Low risk | Low risk | Low risk |
| Yang 2020 | Serious risk | Serious risk | Moderate risk | Low risk | Low risk | Low risk | Low risk | Low risk |
| Fosbol 2020 | Moderate risk | Low risk | Low risk | Low risk | Moderate risk | Low risk | Low risk | Low risk |
| Li 2020 | Moderate risk | Moderate risk | Low risk | Low risk | Low risk | Low risk | Low risk | Low risk |
| Zhou 2020 | Low risk | Low risk | Low risk | Low risk | Low risk | Low risk | Low risk | Low risk |
| Andrea 2020 | Moderate risk | Low risk | Moderate risk | Low risk | Low risk | Low risk | Low risk | Moderate risk |
| Matsuzawa 2020 | Low risk | Low risk | Low risk | Low risk | Low risk | Low risk | Low risk | Low risk |
| Ran 2020 | Low risk | Low risk | Low risk | Low risk | Low risk | Low risk | Low risk | Low risk |
| Lee 2020 | Moderate risk | Low risk | Low risk | Moderate risk | Low risk | Low risk | Low risk | Low risk |
| Seo 2020 | Low risk | Low risk | Low risk | Low risk | Low risk | Low risk | Low risk | Low risk |
| Trifiro 2020 | Moderate risk | Low risk | Low risk | Moderate risk | Low risk | Low risk | Low risk | Low risk |
| Felice 2020 | Low risk | Low risk | Low risk | Low risk | Low risk | Low risk | Low risk | Low risk |
| Khan 2020 | Serious risk | Moderate risk | Low risk | Low risk | Low risk | Low risk | Low risk | Serious risk |
| Soleimani 2020 | Moderate risk | Low risk | Moderate risk | Low risk | Low risk | Low risk | Low risk | Low risk |
| Pan 2020 | Moderate risk | Moderate risk | Low risk | Low risk | Low risk | Low risk | Low risk | Low risk |
| Wang 2020 | Moderate risk | Moderate risk | Low risk | Low risk | Low risk | Low risk | Low risk | Low risk |
| [Peng 2020] | Moderate risk | Moderate risk | Low risk | Low risk | Low risk | Low risk | Low risk | Low risk |
| [Huang 2020] | Low risk | Low risk | Low risk | Low risk | Low risk | Low risk | Low risk | Low risk |
| [Zhuang 2020] | Low risk | Low risk | Low risk | Low risk | Low risk | Low risk | Low risk | Low risk |

**Supplementary Table: Assessment results of risk of bias by ROBINS-tool**
