# Supplementary material for: Influence of angiotensin converting enzyme inhibitors/angiotensin receptor blockers on the risk of all‐cause mortality and other clinical outcomes in patients with confirmed COVID‐19: A systemic review and meta‐analysis
Source: J Clin Hypertens (Greenwich). 2021 Jul 28;23(9):1651–63. doi: 10.1111/jch.14329 (PMC8420264; doi:10.1111/jch.14329)
Supplement: Supplementary file 3 — Supporting material [file JCH-23-1651-s002.docx]

**Appendix file**

**Contents**

Table 1. Duration of hospital stay

Figure 1. Meta-analysis of mortality event on whole population

Figure 2. Meta-analysis of crude OR on mortality on whole population

Figure 3. Meta-analysis of adjusted OR on mortality on whole population

Figure 4. Meta-analysis of HR on mortality on whole population

Figure 5. Meta-analysis of Crude OR for ventilation

Figure 6. Meta-analysis of adjusted OR for ventilation

Figure 7. Meta-analysis of crude OR for heart failure

Figure 8. Meta-analysis of adjusted OR for heart failure

Figure 9. Meta-analysis of mortality events on hypertension subgroup analysis

Figure 10. Meta-analysis of mortality crude OR on hypertension subgroup analysis

Figure 11. Meta-analysis of mortality adjusted OR on hypertension subgroup analysis

Figure 12. Meta-analysis of mortality HR on hypertension subgroup analysis

Figure 13. Meta-analysis of mortality crude OR on age difference subgroup analysis

Table 2. Age difference of ACEI/ARB use group and non-use group

Figure 14. Meta-analysis of mortality events on ACEI/ARBs subgroup analysis

Figure 15. Meta-analysis of mortality crude OR on ACEI/ARBs subgroup analysis

Figure 16. Meta-analysis of mortality adjusted OR on ACEI/ARBs subgroup analysis

Figure 17. Meta-analysis of mortality HR on ACEI/ARBs subgroup analysis

Figure 18. Meta-analysis of mortality event on ACEI users vs ARB users

Figure 19. Meta-analysis of crude mortality on ACEI users vs ARB users

Figure 20. Sensitivity analysis based on risk of bias after high risk study out - mortality events

Figure 21. Sensitivity analysis based on risk of bias after high risk study out - mortality crude OR

Figure 22. Sensitivity analysis based on risk of bias after high risk study out - mortality adjusted OR

Figure 23. Sensitivity analysis based on risk of bias after high risk study out - mortality HR

Figure 24. Sensitivity analysis in hypertension subgroup based on risk of bias after high risk study out - mortality events

Figure 25. Sensitivity analysis in hypertension subgroup based on risk of bias after high risk study out - mortality crude OR

Figure 26. Sensitivity analysis in hypertension subgroup based on risk of bias after high risk study out - mortality adjusted OR

Figure 27. Sensitivity analysis in hypertension subgroup based on risk of bias after high risk study out - mortality HR

Supplementary Tale 1 Duration of hospital stay

| Author | ACEI/ARB user | No user |
| --- | --- | --- |
| Richardson |  |  |
| Discharge group，ACEI user | 3.7 (2.2-6.2) | 3.7 (2.8-7.3) |
| Dead group，ACEI user | 6.5 (3.5-9.5) | 6.5 (2.1-6.9) |
| Discharge group，ARB user | 4.1 (2.4-6.5) | 3.7 (2.8-7.3) |
| Dead group，ARB user | 5 (2.2-7.7) | 6.5 (2.1-6.9) |
| Gao | 42 (33-51) | 40 (31-49) |
| Wang | 17 (13-21) | 17 (13-21) |
| Felice |  |  |
| ACEI user | 9.1 ± 5.4 | 11 ± 9.1 |
| ARB user | 8.5 ± 4.5 | 11 ± 9.1 |
| Li | 19 (13-27) | 19 (11-27) |
| Soleimani | 6.0 (3-10) | 4.0 (2-6) |
| Khan (only ACEI) | 14 (8-22) | 17 (10-29) |

Supplementary Figure 1. Meta-analysis of mortality event on whole population

**
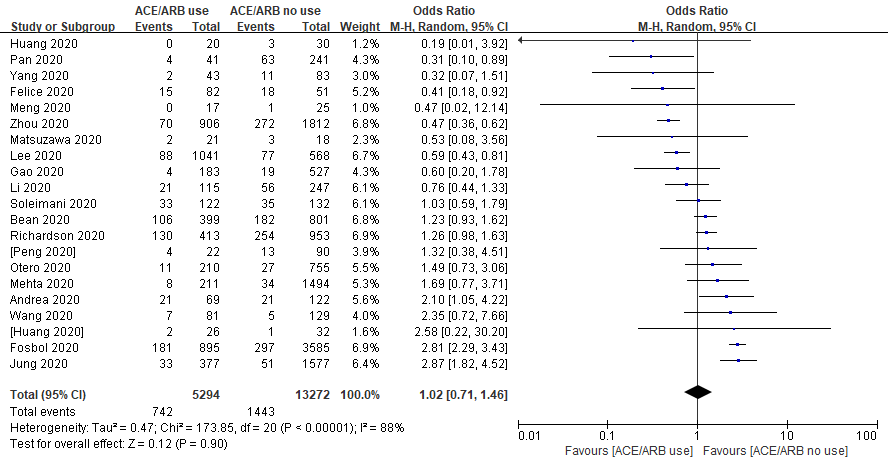
**

**
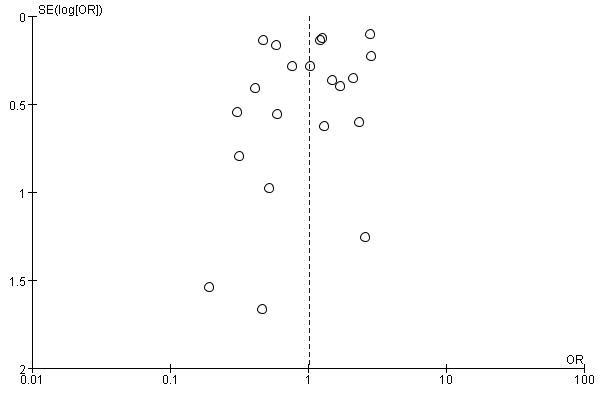
**

Supplementary Figure 2. Meta-analysis of crude OR on mortality on whole population


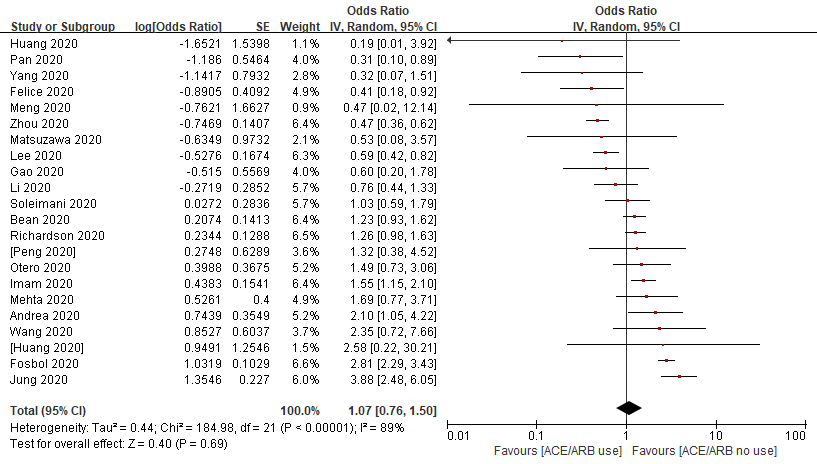


**
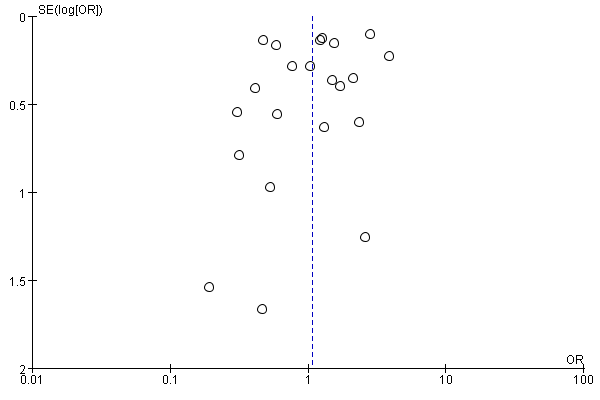
**

Supplementary Figure 3. Meta-analysis of adjusted OR on mortality on whole population

**
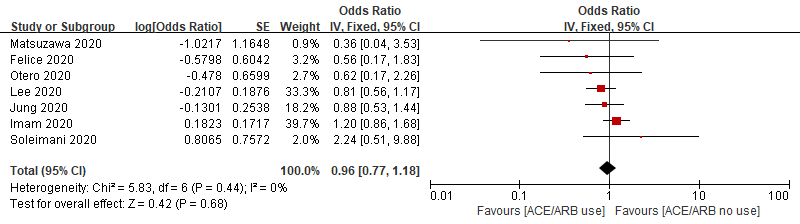
**

Supplementary Figure 4. Meta-analysis of HR on mortality on whole population

**
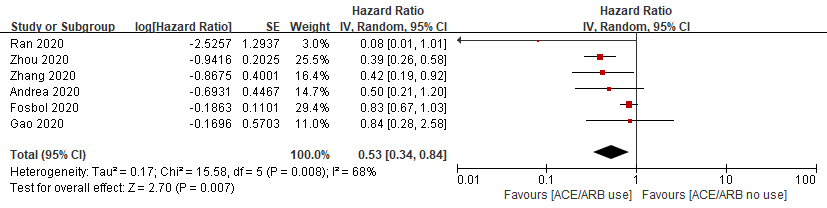
**

Supplementary Figure 5. Meta-analysis of Crude OR for ventilation


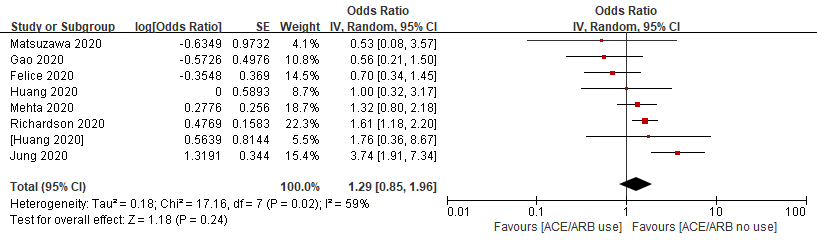


Supplementary Figure 6. Meta-analysis of adjusted OR for ventilation

**
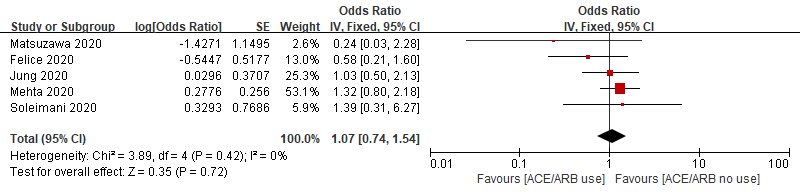
**

Supplementary Figure 7. Meta-analysis of crude OR for heart failure

**
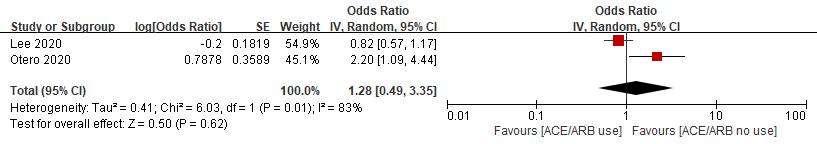
**

Supplementary Figure 8. Meta-analysis of adjusted OR for heart failure

**
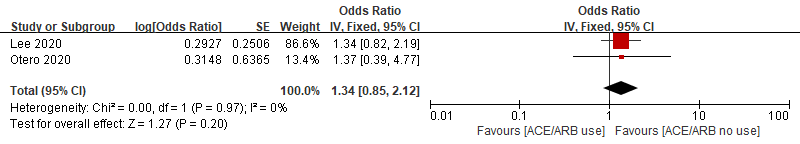
**

Supplementary Figure 9. Meta-analysis of mortality events on hypertension subgroup analysis

**
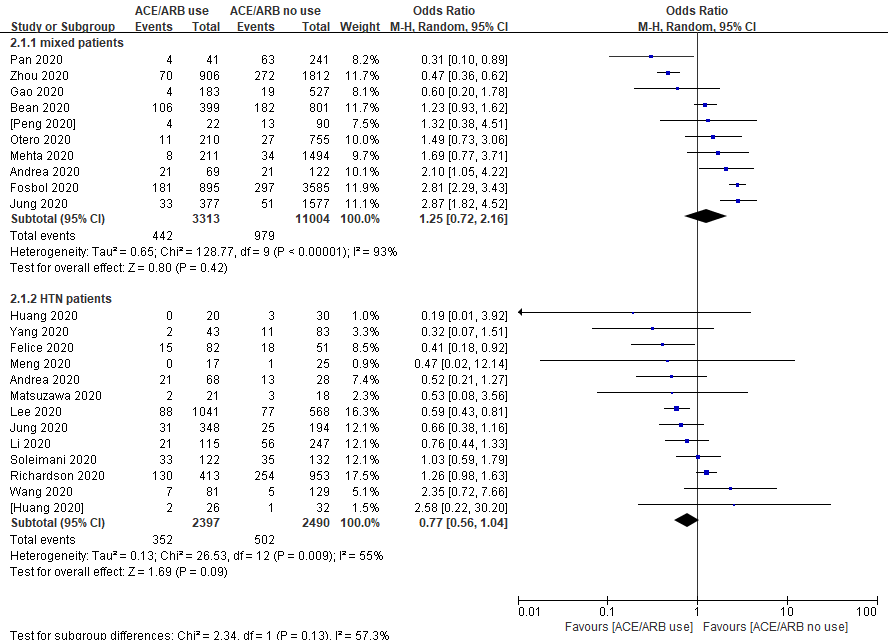
**

Supplementary Figure 10. Meta-analysis of mortality crude OR on hypertension subgroup analysis

**
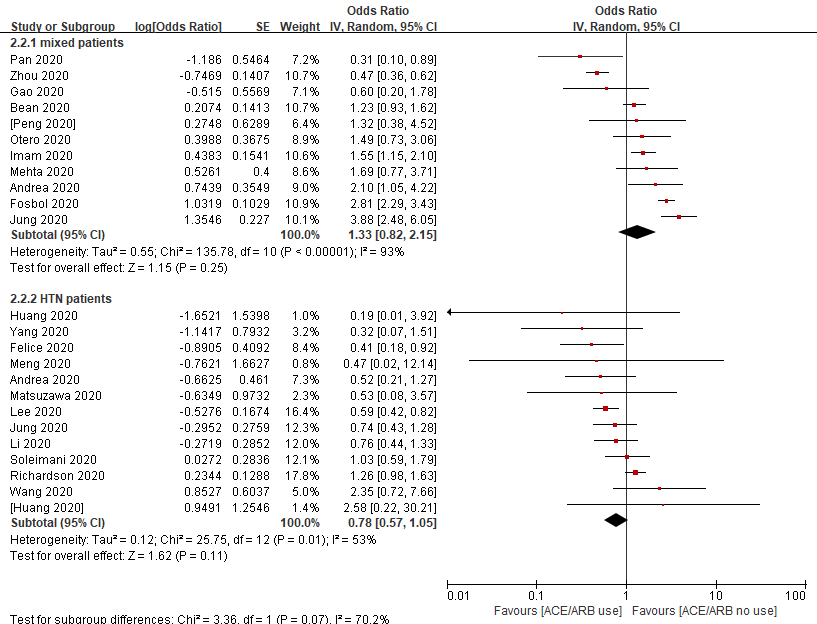
**

Supplementary Figure 11. Meta-analysis of mortality adjusted OR on hypertension subgroup analysis

**
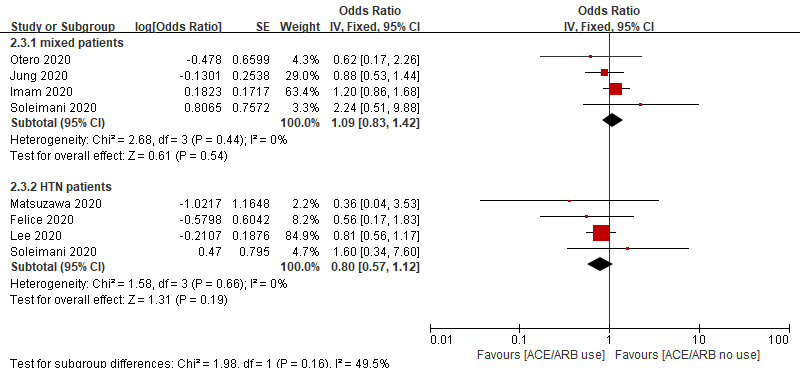
**

Supplementary Figure 12. Meta-analysis of mortality HR on hypertension subgroup analysis

**
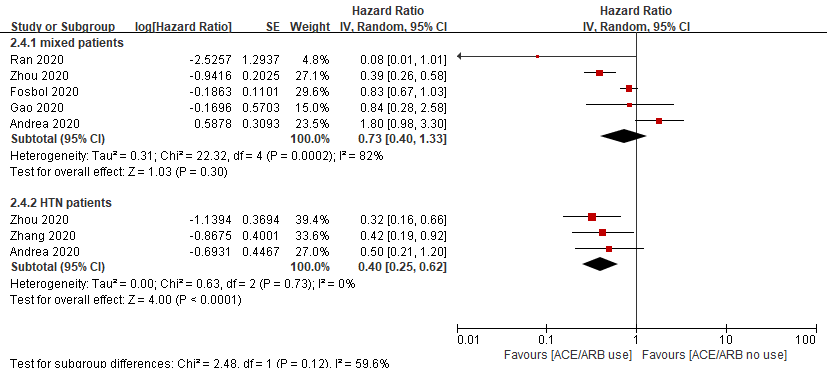
**

Supplementary Figure 13. Meta-analysis of mortality crude OR on age-difference subgroup analysis

**
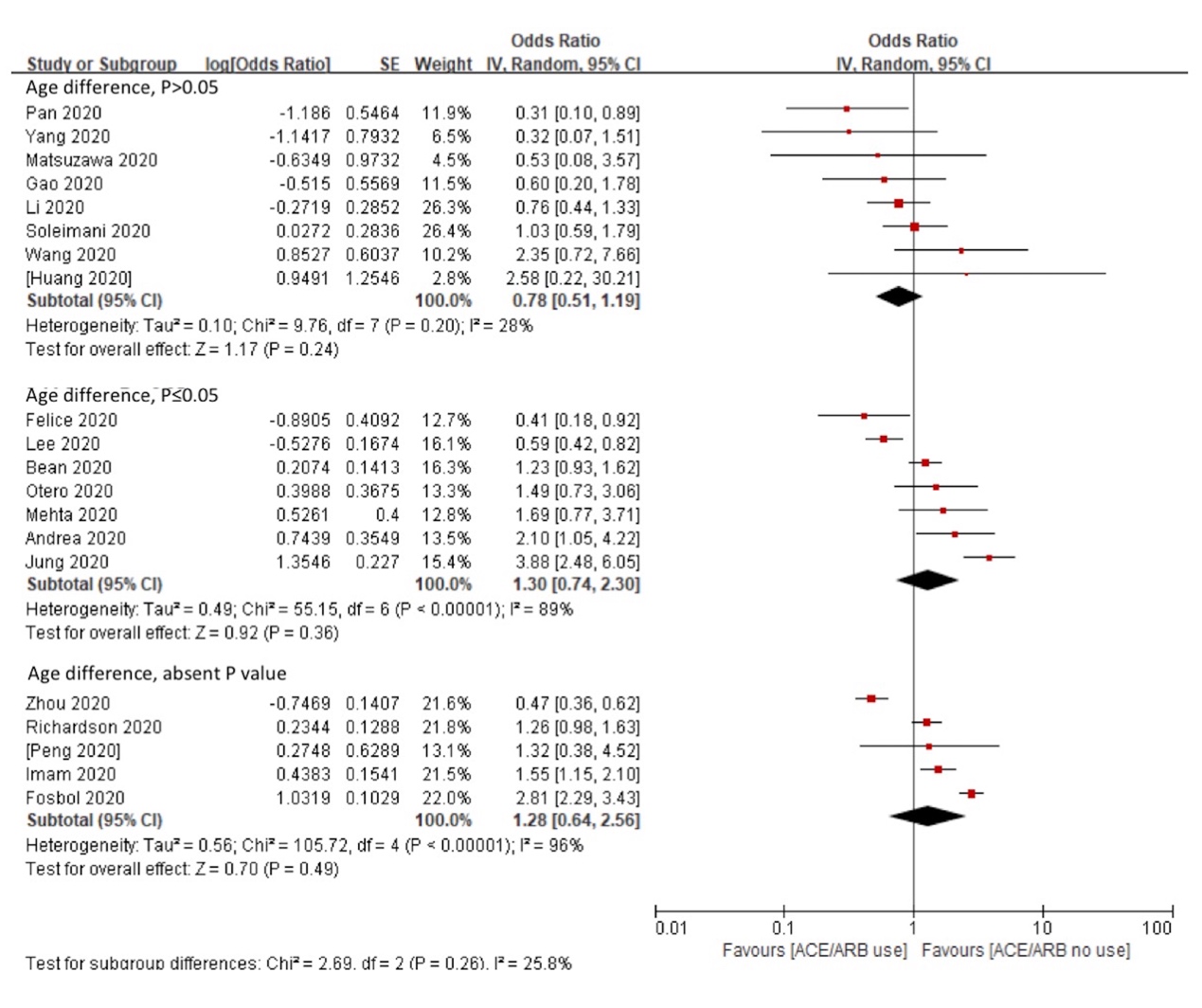
**

Supplementary Table 2. Age difference of ACEI/ARB use group and non-use group

| **Study** | **ACEI/ARB user** | **Non-user** | **P value** |
| --- | --- | --- | --- |
| **Age difference, P>0.05** | | | |
| Pan 2020 | 70 (63 - 76) | 69 (62 - 76) | 0.889 |
| Yang 2020 | 65 (57 - 72) | 67 (62 - 75) | 0.460 |
| Matsuzawa 2020 | 71 (11) | 72 (12) | 0.86 |
| Gao 2020 | 64.84 (11.19) | 62.64 (11.00) | 0.919 |
| Li 2020 | 65 (57 - 73) | 67 (60 - 75) | 0.22 |
| Soleimani 2020 | 68.0 (11.7) | 64.9 (13.8) | 0.051 |
| Wang 2020 | 68 (61.5 - 76) | 66 (59 - 72.5) | 0.107 |
| [Huang 2020] | 64 (50, 72) | 64 (57, 69) | 0.969 |
| **Age difference, P<0.05** | | | |
| Felice 2020 | ACEI 73.1(11.5) | 76.2 (11.9) | 0.023 |
|  | ARB 69 (13.4) |  |  |
| Lee 2020 | 66.8 (14.7) | 69.3 (14.9) | <0.001 |
| Bean 2020 | 73.02 (13.46) | 65.45 (18.1) | <0.001 |
| Otero 2020 | 72.1 (13.2) | 56.0 (20.5) | <0.001 |
| Mehta 2020 | ACEI 63 (15) | 53 (19) | <0.001 |
|  | ARB 65 (13) |  |  |
| Khan 2020 | 74.61 (13.33) | 66.22 (12.25) | 0.006 |
| Jung 2020 | 62.5 (14.7) | 41.5 (16.6) | <0.001 |
| **Age difference, P absent** | | | |
| Zhou 2020 | ACEI 65 (59 - 71) | NR | P absent |
|  | ARB 64 (56 - 72) |  |  |
| Richardson 2020 | NR | NR | P absent |
| Imam 2020 | NR | NR | P absent |
| Andrea 2020 | NR | NR | P absent |
| Fosbol 2020 | 72.8 (61 - 81) | 50.1 (37.2 - 64.5) | P absent |
| [Peng 2020] | NR | NR | P absent |

Abbreviation: ACEI: angiotensin converting enzyme inhibitor; ARB: angiotensin receptor blocker; NR, no report.

[] means Chinese articles.

Supplementary Figure 14. Meta-analysis of mortality events on ACEI/ARBs subgroup analysis

**
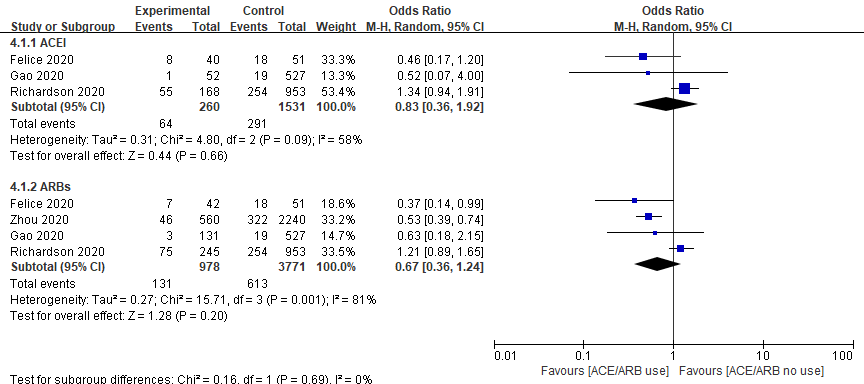
**

Supplementary Figure 15. Meta-analysis of mortality crude OR on ACEI/ARBs subgroup analysis

**
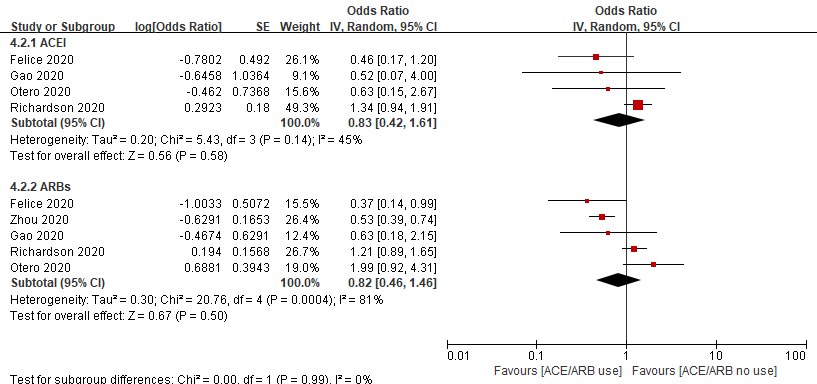
**

Supplementary Figure 16. Meta-analysis of mortality adjusted OR on ACEI/ARBs subgroup analysis

**
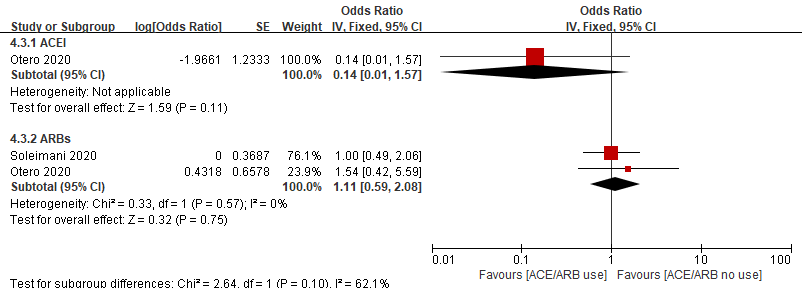
**

Supplementary Figure 17. Meta-analysis of mortality HR on ACEI/ARBs subgroup analysis

**
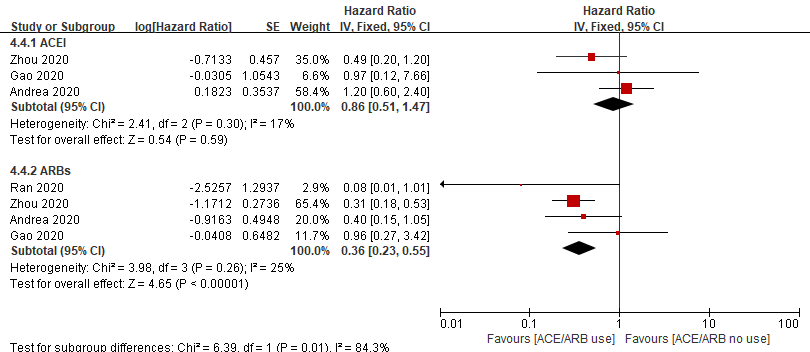
**

Supplementary Figure 18. Meta-analysis of mortality event on ACEI users vs ARB users

**
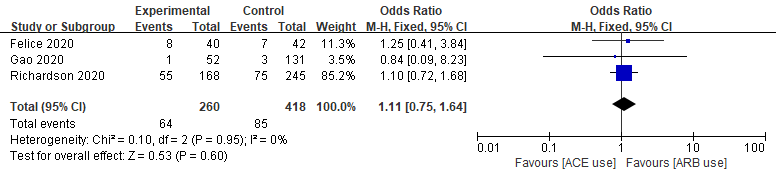
**

Supplementary Figure 19. Meta-analysis of crude mortality on ACEI users vs ARB users

**
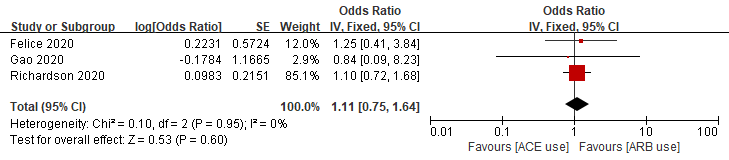
**

Supplementary Figure 20. Sensitivity analysis in whole population based on risk of bias after high risk study out - mortality events

**
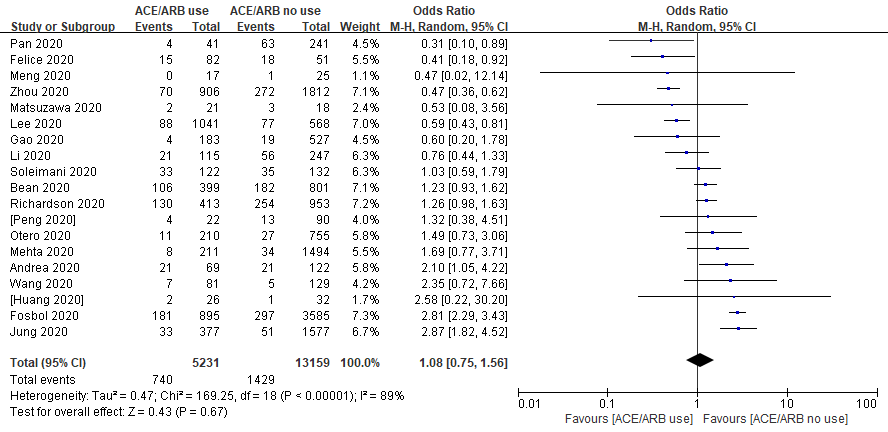
**

Supplementary Figure 21. Sensitivity analysis in whole population based on risk of bias after high risk study out - mortality crude OR

**
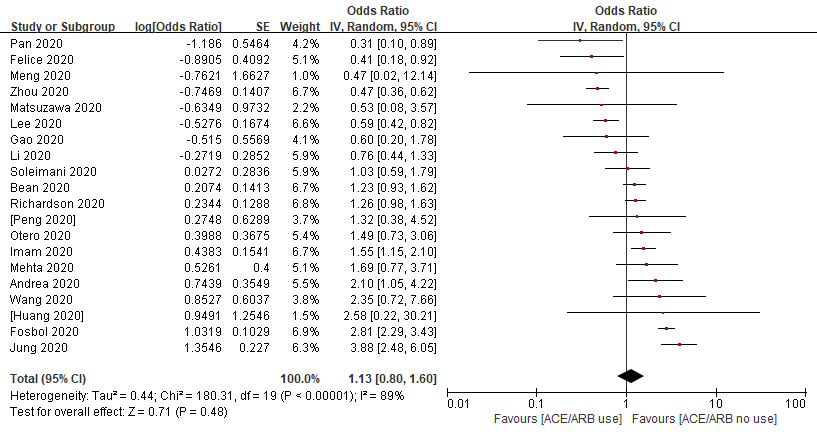
**

Supplementary Figure 22. Sensitivity analysis in whole population based on risk of bias after high risk study out - mortality adjusted OR

**
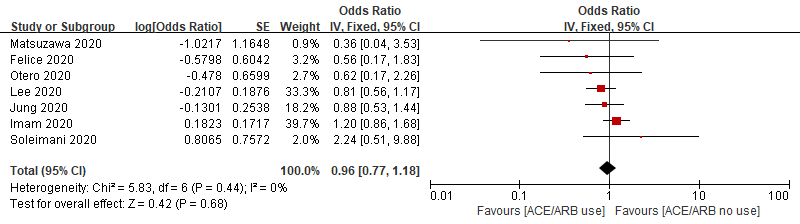
**

Supplementary Figure 23. Sensitivity analysis in whole population based on risk of bias after high risk study out - mortality HR

**
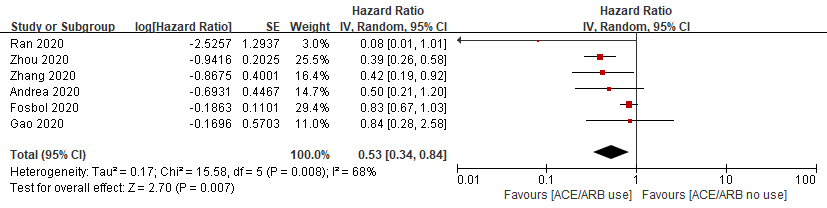
**

Supplementary Figure 24. Sensitivity analysis in hypertension subgroup based on risk of bias after high risk study out - mortality events

**
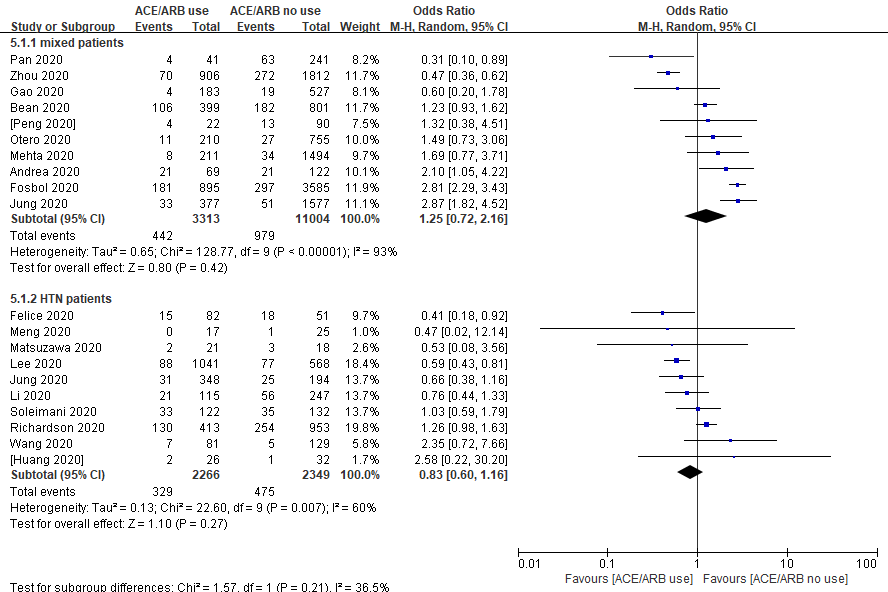
**

Supplementary Figure 25. Sensitivity analysis in hypertension subgroup based on risk of bias after high risk study out - mortality crude OR

**
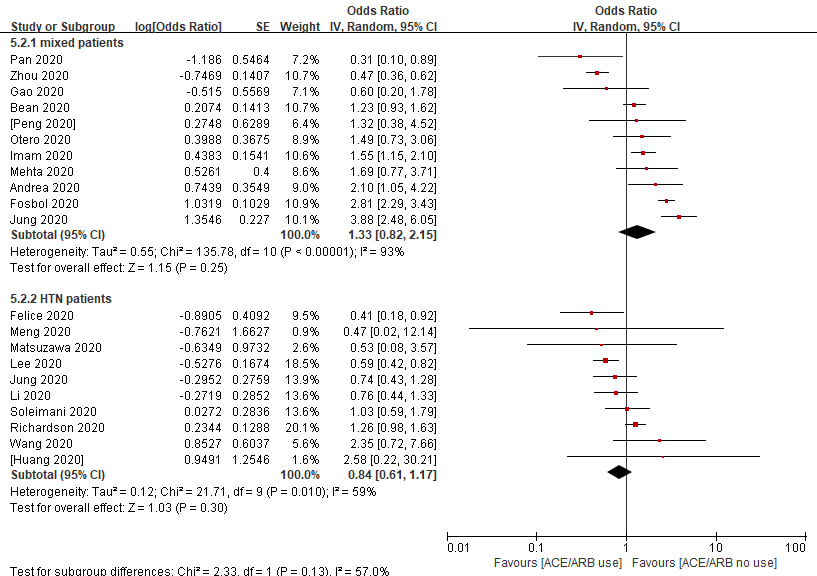
**

Supplementary Figure 26. Sensitivity analysis in hypertension subgroup based on risk of bias after high risk study out - mortality adjusted OR

**
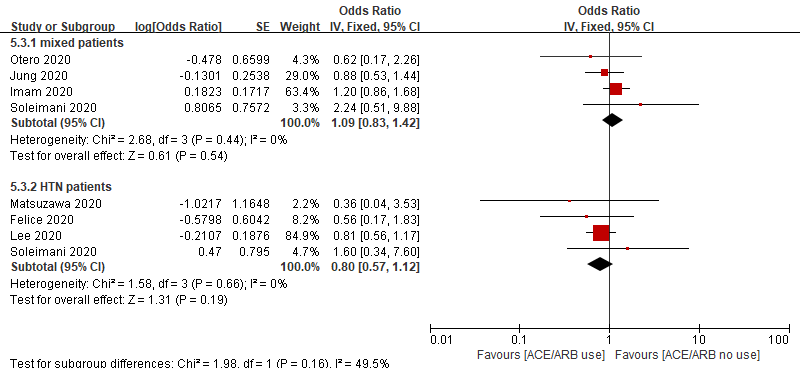
**

Supplementary Figure 27. Sensitivity analysis in hypertension subgroup based on risk of bias after high risk study out - mortality HR

**
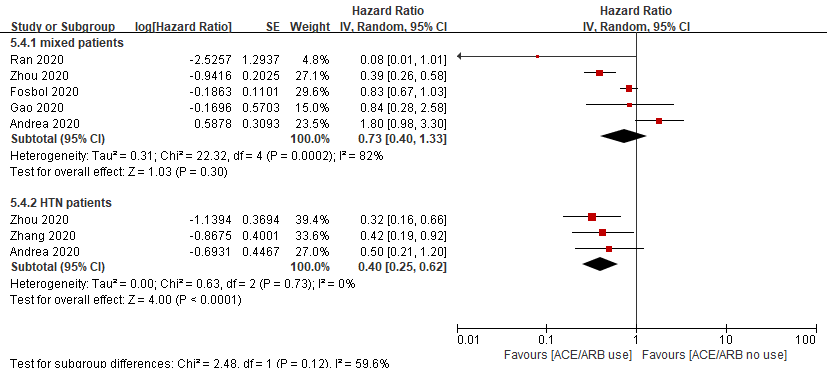
**
